# CRISPRi-based radiation modifier screen identifies long non-coding RNA therapeutic targets in glioma

**DOI:** 10.1186/s13059-020-01995-4

**Published:** 2020-03-31

**Authors:** S. John Liu, Martina Malatesta, Brian V. Lien, Parna Saha, Shivani S. Thombare, Sung Jun Hong, Leslie Pedraza, Mark Koontz, Kyounghee Seo, Max A. Horlbeck, Daniel He, Harjus S. Birk, Miten Jain, Hugh E. Olsen, Mark Akeson, Jonathan S. Weissman, Michelle Monje, Nalin Gupta, David R. Raleigh, Erik M. Ullian, Daniel A. Lim

**Affiliations:** 1 Department of Neurological Surgery, University of California, San Francisco, CA 94143 USA; 2 Eli and Edythe Broad Center of Regeneration Medicine and Stem Cell Research, University of California, San Francisco, CA 94143 USA; 3 Department of Radiation Oncology, University of California, San Francisco, CA 94143 USA; 4 San Francisco Veterans Affairs Medical Center, San Francisco, CA USA; 5 Developmental and Stem Cell Biology Graduate Program, University of California, San Francisco, CA 94143 USA; 6 Department of Ophthalmology, University of California, San Francisco, CA 94143 USA; 7 Department of Cellular and Molecular Pharmacology, University of California, San Francisco, CA 94143 USA; 8 Howard Hughes Medical Institute, University of California, San Francisco, CA 94143 USA; 9 California Institute for Quantitative Biomedical Research, University of California, San Francisco, CA 94143 USA; 10 Center for RNA Systems Biology, University of California, San Francisco, CA 94143 USA; 11 UC Santa Cruz Genomics Institute, University of California, Santa Cruz, CA 95064 USA; 12 Department of Neurology and Neurological Sciences, Stanford University, Stanford, CA 94305 USA

**Keywords:** CRISPRi, lncRNA, Organoids, Radiation, Glioma, Cancer therapy

## Abstract

**Background:**

Long non-coding RNAs (lncRNAs) exhibit highly cell type-specific expression and function, making this class of transcript attractive for targeted cancer therapy. However, the vast majority of lncRNAs have not been tested as potential therapeutic targets, particularly in the context of currently used cancer treatments. Malignant glioma is rapidly fatal, and ionizing radiation is part of the current standard-of-care used to slow tumor growth in both adult and pediatric patients.

**Results:**

We use CRISPR interference (CRISPRi) to screen 5689 lncRNA loci in human glioblastoma (GBM) cells, identifying 467 hits that modify cell growth in the presence of clinically relevant doses of fractionated radiation. Thirty-three of these lncRNA hits sensitize cells to radiation, and based on their expression in adult and pediatric gliomas, nine of these hits are prioritized as lncRNA Glioma Radiation Sensitizers (*lncGRS*). Knockdown of *lncGRS-1*, a primate-conserved, nuclear-enriched lncRNA, inhibits the growth and proliferation of primary adult and pediatric glioma cells, but not the viability of normal brain cells. Using human brain organoids comprised of mature neural cell types as a three-dimensional tissue substrate to model the invasive growth of glioma, we find that antisense oligonucleotides targeting *lncGRS-1* selectively decrease tumor growth and sensitize glioma cells to radiation therapy.

**Conclusions:**

These studies identify *lncGRS-1* as a glioma-specific therapeutic target and establish a generalizable approach to rapidly identify novel therapeutic targets in the vast non-coding genome to enhance radiation therapy.

## Background

The human genome produces many thousands of lncRNAs [1–3]—transcripts longer than 200 nucleotides that do not encode for proteins—and certain lncRNAs play key roles in the pathogenesis of cancer [4–7]. LncRNAs exhibit highly cell type-specific expression and function [1, 8], making this class of transcripts attractive for targeted cancer therapy. However, it is currently not possible to predict which of these non-coding transcripts would be effective therapeutic targets, let alone those that could sensitize cancer cells to radiation. To rapidly develop lncRNAs as a class of targets for cancer therapy, systematic functional screens are necessary.

CRISPR-based technologies have enabled genome-scale screens of gene function in mammalian cells [8–16]. These screening methods have been valuable to the identification of genes—non-coding in addition to coding—that are essential for various cellular phenotypes. However, whether such screen-identified hits can increase the efficacy of ionizing radiation—a critical adjunctive cancer therapy for many malignancies—has not been systematically studied at large scale.

Malignant glioma—a primary cancer of the central nervous system (CNS)—is a fatal diagnosis for most patients [17]. Despite surgery and adjuvant therapy such as fractionated radiation, adults with glioblastoma (GBM) have a median survival of only 14 months [18, 19]. In children, the most common malignant glioma is diffuse intrinsic pontine glioma (DIPG). DIPG is primarily treated with radiotherapy, but median survival is only 9–10 months, and few patients survive more than 2 years after diagnosis [20–22]. While radiation is a critical component of the treatment of both adult and pediatric malignant gliomas by reducing tumor growth [19, 23, 24], the toxicity of radiation to normal brain cells limits the total dose that can be delivered, and glioma cells that survive radiation lead to tumor recurrence. Thus, therapeutics that increase the efficacy of radiation without reducing the viability of normal brain cells would be of critical clinical benefit, complementing the current standard-of-care for nearly all patients with malignant gliomas.

Organoids are miniature three-dimensional (3D) representations of their in vivo counterpart organs, and organoid-based models of cancer are emerging as a useful platform for the evaluation of therapeutics [25]. Human brain organoids have been generated from pluripotent stem cell (PSC) populations, mimicking early stages of fetal brain development [26–28]. Such embryonic brain organoids have been useful to study the genetic mutations that cause GBM [29] and can also serve as a 3D tissue substrate for the growth of glial tumors [30]. However, embryonic brain organoids do not closely represent the mature brain tissue of glioma patients, limiting their utility for assessing potential drug toxicity to normal adult brain cells.

Here, we developed a radiation modifier screen using CRISPRi to identify specific lncRNAs that sensitize glioma cells to radiotherapy. In this screen of 5689 lncRNA loci, 467 hits were found to modify cell growth in the presence of radiation. Thirty-three of these hits sensitized cells to clinically relevant doses of fractionated radiation, and based on their expression in adult and pediatric glioma, nine of these hits were prioritized as lncRNA Glioma Radiation Sensitizers (*lncGRS*). Knockdown of *lncGRS-1* (*CTC-338 M12.4* located on chromosome 5 q35.3) inhibited the growth of primary adult and pediatric glioma cells, but the viability of normal brain cells was not harmed. While *lncGRS-1* is primate-conserved, this lncRNA does not exist in rodents, making traditional in vivo mouse models of glioma suboptimal for assessing potential toxicity of *lncGRS-1* knockdown in normal brain tissue. We therefore developed a novel human brain organoid model of malignant glioma. To mimic the brains of patients, we assembled “mature” human brain organoids (MBOs) from mature neural cell types derived from induced pluripotent stem cells (iPSCs), and human glioma cells grew invasively within these 3D tissues. Furthermore, antisense oligonucleotides (ASOs) targeting *lncGRS-1* selectively decreased tumor growth and sensitized glioma cells to radiation therapy. These studies identify *lncGRS-1* as a glioma-specific therapeutic target and establish a generalizable approach to rapidly identify novel therapeutic targets in the vast non-coding genome.

## Results

### A CRISPRi platform for radiotherapy sensitization in a glioma cell culture model

To systematically identify lncRNAs as potential therapeutic targets that sensitize malignant glioma to radiotherapy, we developed a radiation modifier screen using CRISPRi for gene knockdown. CRISPRi represses transcription via the recruitment of catalytically “dead” Cas9 protein fused to the KRAB repressor (dCas9-KRAB), which is targeted to transcriptional start sites (TSS) by a single guide RNA (sgRNA) [11, 31, 32]. For the screen, we used a workhorse GBM cell line (U87) engineered to stably express dCas9-KRAB (U87-dCas9-KRAB) to identify hits for subsequent study in patient-derived cultures of pediatric and adult forms of malignant glioma.

**Fig. 1:**
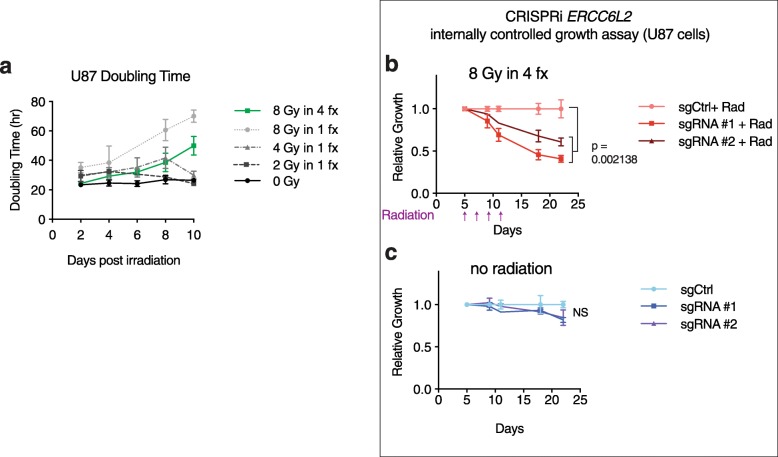
A glioma cell culture model for identifying radiation sensitizers. **a** Doubling time of U87 GBM cells in culture after treatment with different amounts of single-dose or fractionated radiation. Fractionated (fx) radiation (8 Gy in 4 fx) was delivered in 2 Gy doses every other day (*n* = 3 biological replicates per condition; error bar = SD). **b**, **c** Internally controlled growth assays for U87 cells evaluating CRISPRi knockdown of *ERCC6L2* with (**b**) and without (**c**) radiation (*n* = 3 biological replicates per condition; error bar = SD; two-tailed Student’s *t* test of end points; radiation delivery timepoints indicated below the *x*-axis)

First, we sought to determine a radiation dose and delivery schedule in U87-dCas9-KRAB cells that enables the discovery of radiation-effect modifiers. For the treatment of human GBM patients, the total radiation dose is typically delivered in ~ 2 Gy daily fractions [18, 19]. When 2 Gy was delivered to U87-dCas9-KRAB cells as a single dose, cell proliferation transiently decreased but returned to normal after 8–10 days (Fig. 1a). A single dose of 4 or 8 Gy had correspondingly stronger and more prolonged inhibitory effects upon cell proliferation (Fig. 1a), and RNA-seq analysis revealed dose-dependent gene expression changes including induction of the p53 pathway and repression of DNA replication (Additional file 1: Fig. S1a). However, these higher single doses exceed those typically used for patients with malignant glioma. Thus, we also tested a total radiation dose of 8 Gy delivered in 2 Gy fractions given every other day (8 Gy in 4 fractions). This 8 Gy fractionated dose increased the cell doubling time by approximately 2-fold, which is an effect size approximating LD_50_ (Fig. 1a), which allows optimal discovery of both synergistic and buffering screen hits [33], while still utilizing a clinically relevant fractional dose of 2 Gy.

We next tested whether U87-dCas9-KRAB cells treated with 8 Gy fractionated radiation can reveal radiation sensitization effects with CRISPRi gene targeting. The inhibition of DNA repair pathways is known to potentiate the therapeutic effects of radiation in cancer cells [34, 35]. In internally controlled growth assays [8, 11], two distinct CRISPRi sgRNAs targeting the DNA damage repair gene *ERCC6L2* [36, 37] reduced cell growth in U87 cells treated with 8 Gy fractionated radiation (Fig. 1b). Without radiation treatment, CRISPRi targeting of *ERCC6L2* did not reduce cell growth (Fig. 1c). Thus, CRISPRi targeting of the DNA repair gene *ERCC6L2* produces a strong radiation sensitization effect in this in vitro model of fractionated radiotherapy.

### CRISPRi screen identifies lncRNA Glioma Radiation Sensitizer hits

**Fig. 2:**
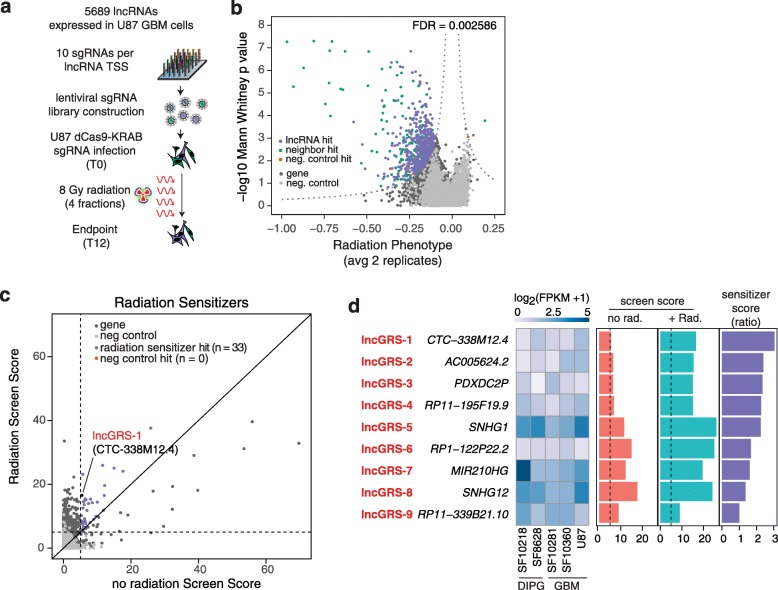
CRISPRi radiation modifier screen in glioma cells. **a** Experimental design. Eight Gy radiation was delivered in 4 fractions throughout the screen. **b** Volcano plot of radiation growth phenotypes (average of two replicate screens) for the top 3 sgRNAs targeting each lncRNA TSS (*x*-axis) and negative log_10_(Mann-Whitney *U p* value) of all sgRNAs for a given gene at T12 as compared to T0 (*y*-axis). Screen threshold was determined by the product of the *x*- and *y*-axes that resulted in an empirical FDR of 0.0025. Neighbor hits are defined as any lncRNA hit with an expressed protein coding gene within 1 kb of the lncRNA TSS. Phenotype refers to the relative log2 enrichment of barcodes in the final timepoint divided by the enrichment of barcodes at the initial timepoint [8, 11]. **c** Comparison of screen scores, defined as the average phenotype of the top three sgRNAs against a given gene multiplied by the negative log10(Mann-Whitney *U p* value) for that gene, from screens conducted without radiation (*x*-axis) with scores from screens conducted with radiation (*y*-axis), with thresholds set at 5 (FDR = 0.0025). Thirty-three lncRNA hits had radiation screen scores greater than no radiation screen scores. *CTC-338 M12.4* (*lncGRS-1*) is indicated. **d** LncRNA expression across 2 DIPG and 3 GBM cultures (subpanel 1), no radiation screen scores (subpanel 2), radiation screen scores (subpanel 3), and sensitizer scores (subpanel 4) for each of the 9 *lncGRS* candidates. Sensitizer score is defined as the ratio of the radiation modifier screen score in irradiated cells to the growth screen score in non-irradiated cells

Although disruption of known DNA repair pathways is an intriguing avenue for novel therapeutics, accumulation of unrepaired DNA damage may paradoxically increase cancer risk [38, 39]. The human genome produces thousands of lncRNAs that represent a large set of novel potential therapeutic targets. We screened the function of 5689 lncRNAs expressed in human glioma by leveraging our CRISPRi Non-Coding Library [8], selecting 10 sgRNAs for each lncRNA TSS and cloning this pool of 56,890 sgRNAs into lentiviral vectors along with 1202 non-targeting control sgRNAs (Fig. 2a). We used this lentiviral sgRNA library to infect two replicates of U87-dCas9-KRAB cultures, selected for infected cells with puromycin, treated cultures with 8 Gy fractionated radiation, then continued cell propagation for a total of 12 days. The proportion of sgRNA positive cells remained stable throughout the screen, indicating that CRISPRi targeting does not exhibit non-specific toxicity even after radiation treatment (Additional file 1: Fig. S2a).

Targeted next generation sequencing of sgRNA barcodes performed at the beginning and end of the screen (~ 7.1 cell doublings) identified 652 loci that modified cell growth and proliferation in the presence of radiation as compared to the effect of radiation alone (FDR = 0.25%). One hundred eighty-five of these lncRNA TSS hits were within 1 kb of an expressed protein-coding gene and therefore conservatively labeled as “neighbor hits,” with the 1 kb window around the TSS being based on analyses of CRISPRi mechanism and maximum effective distance for knockdown [8, 11]. These neighbor hits were removed from further analysis, leaving 467 “lncRNA hits” that modified the propagation of radiation-treated U87 cells (Fig. 2b; Additional file 2: Table S1). Interestingly, CRISPRi-mediated knockdown of the lncRNA *PVT1* protected cells against the effect of radiation (Additional file 1: Fig. S2b), consistent with *PVT1* acting as a negative regulator of *MYC* [40]. The other 466 lncRNA hits negatively affected cell culture growth when combined with radiation.

**Fig. 3:**
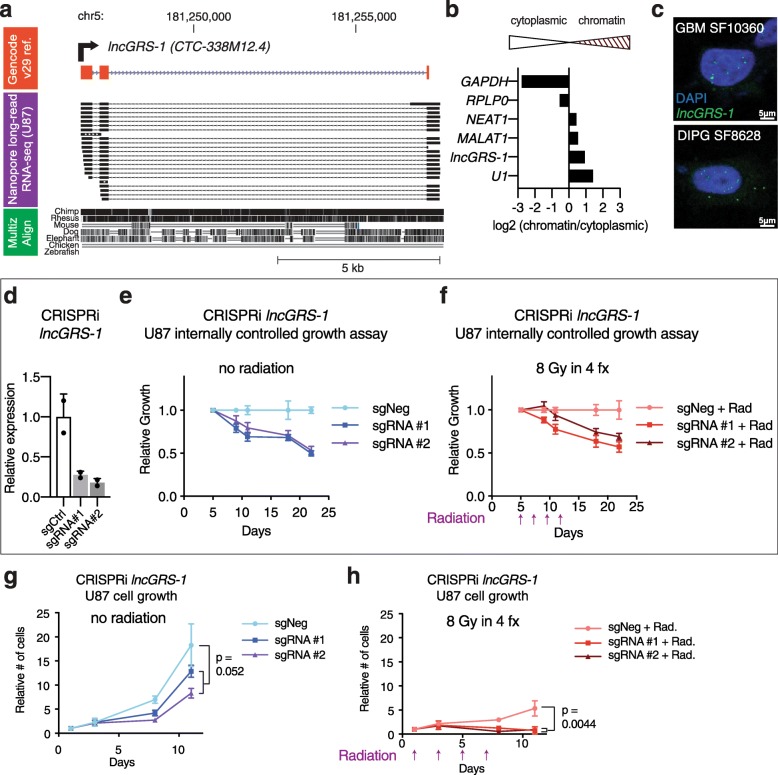
*lncGRS-1* is a primate-conserved radiation sensitizer target in glioma. **a** Nanopore direct RNA-seq spliced reads aligned to the *lncGRS-1* gene body in U87 cells, with GENCODE v29 transcript models of *lncGRS-1* (*CTC-338 M12.4*) and multiz alignment for conservation. **b** Subcellular fractionation followed by qPCR of transcripts in U87 cells. **c** Single molecule FISH of *lncGRS-1* in GBM SF10360 and DIPG SF8628 primary glioma cells. Scale bar = 5 μm. **d** RT-qPCR for *lncGRS-1* after CRISPRi targeting in U87 (*n* = 2 biological replicates; error bar = SD). **e**, **f** Internally controlled growth assays of U87 cells with CRISPRi knockdown of *lncGRS-1* in the absence (**e**) and presence (**f**) of fractionated radiation. **g**, **h** Cell propagation assay of purified populations of U87 cells with *lncGRS-1* CRISPRi knockdown in the absence (**g**) and presence (**h**) of fractionated radiation. For **e**–**h**, *n* = 2 biological replicates per condition; error bars = SD; two-tailed Student’s *t* test. N.S., not significant. Radiation delivery timepoints indicated below the *x*-axis

To identify lncRNA hits that sensitized cells to the effect of radiation, we compared the screen scores (defined as the average phenotype of the top three sgRNAs against a given gene multiplied by the negative log_10_(Mann-Whitney *U p* value) for that gene) for the radiation modifier screen with the growth screen scores from U87 cells that were not irradiated [8]. Phenotype in these CRISPRi screens refers to the relative log2 enrichment of barcodes in the final timepoint divided by the enrichment of barcodes at the initial timepoint [8, 11]. Thirty three hits identified in both screens had screen scores that were greater in the radiation modifier screen (Fig. 2c). Interestingly, the expression of these 33 sensitizer lncRNA hits tended to be downregulated following exposure to 8 Gy of radiation (Pearson’s *R* = 0.36), which was not observed for the larger set of 467 hits (Pearson’s *R* = 0.079) (Additional file 1: Fig. S2c), suggesting that transcriptional repression following radiation may be a common feature of lncRNA radiosensitizers. Nine of these 33 sensitizer hits were expressed in a panel of various malignant glioma cells including both adult GBM (U87, SF10360, SF10281) and pediatric DIPG (SF8628, SF10218). We ranked these nine hits by a “sensitizer score,” which we defined as the ratio of the radiation modifier screen score in irradiated cells to the growth screen score in non-irradiated cells, and denoted these genes as *lnc*RNA *G*lioma *R*adiation *S*ensitizers (*lncGRS*) 1 to 9 (Fig. 2d). To more accurately survey the transcript structure(s) of these hits, we performed long-read single molecule native RNA sequencing using the Oxford Nanopore PromethION and defined transcript variants and splice boundaries of *lncGRS-1* through *lncGRS-9* (Fig. 3a, Additional file 1: Fig. S3).

### CRISPRi-mediated knockdown of *lncGRS-1* synergizes with fractionated radiation

*lncGRS-1* encodes spliced, poly-adenylated transcripts (687 to 1013 bp) from chromosome 5 (Fig. 3a, Additional file 1: Fig. S3). Although previously annotated (*CTC-338 M12.4*) [41], this lncRNA’s biological function was not known. Cell fractionation analysis (Fig. 3b) and in situ hybridization studies (Fig. 3c, Additional file 1: S4a) revealed *lncGRS-1* to be a nuclear-enriched transcript in glioma cells, including those of patient-derived, primary glioma cultures. Furthermore, *lncGRS-1* transcription is downregulated following radiation (Additional file 1: Fig. S1b).

In internally controlled growth assays of U87 cell growth, CRISPRi-mediated knockdown of *lncGRS-1* with two different, individual sgRNAs (Fig. 3d) reduced cell proliferation by ~ 48% in the absence of radiation (Fig. 3e). Treatment of cells with 8 Gy fractionated radiation alone decreased cell proliferation (Fig. 1a), and *lncGRS-1* knockdown further reduced cell proliferation by ~ 37% relative to radiation alone (Fig. 3f). We also purified U87-dCas9-KRAB cells that express sgRNA against *lncGRS-1* or non-targeting control sgRNAs and studied the growth of these cultures with and without 8 Gy fractionated radiation. Without radiation, sgRNAs against *lncGRS-1* reduced cell proliferation by 42% (Fig. 3g), and radiation alone reduced the proliferation of cells expressing control sgRNAs by 71% (Fig. 3g, h). When combined, *lncGRS-1* knockdown and radiation treatment resulted in a pronounced 95% decrease in proliferation (Fig. 3h), indicating synergy of the two treatments, as the predicted decrease in cell proliferation from an additive effect model is only 83% (*p* = 0.0052; see the “Methods” section).

### CRISPRi- and antisense oligonucleotide-mediated *lncGRS-1* knockdown inhibits the growth of glioma cells from both adult and pediatric patients

**Fig. 4:**
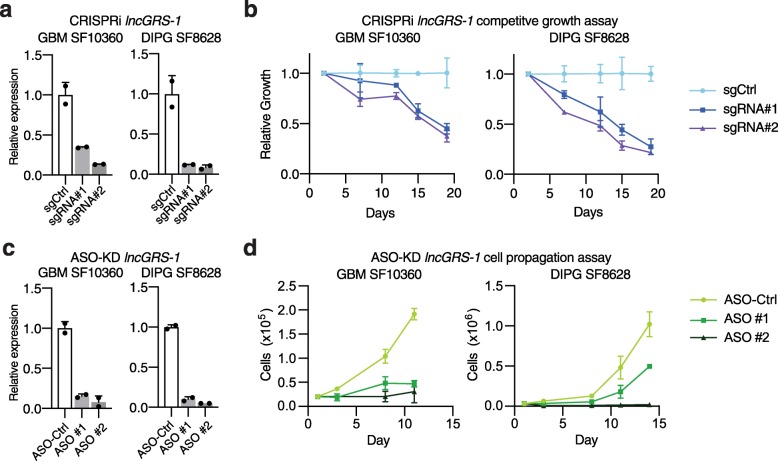
*lncGRS-1* is required for the proliferation of primary, patient-derived glioma cells. **a** RT-qPCR of *lncGRS-1* transcript levels following CRISPRi-mediated knockdown in GBM SF10360 and DIPG SF8628 (*n* = 2 biological replicates per condition; error bars = SD). **b** Internally controlled, growth assays of GBM SF10360 and DIPG SF8628 cells with CRISPRi-mediated knockdown of *lncGRS-1* (*n* = 2 biological replicates per condition; error bars = SD). **c** RT-qPCR of *lncGRS-1* transcript levels following ASO-mediated knockdown of GBM SF10360 and DIPG SF8628 (*n* = 2 biological replicates per condition; error bars = SD). **d** Cell propagation time course of GBM SF10360 and DIPG SF8628 cells with ASO-mediated knockdown of *lncGRS-1*. ASOs were re-transfected at day 7 (*n* = 2 biological replicates per condition; error bars = SD)

We next sought to validate *lncGRS-1* as a potential therapeutic target in patient-derived cultures of malignant glioma. We generated human adult GBM (SF10360) and pediatric DIPG (SF8628) cells stably expressing dCas9-KRAB and studied the effect of CRISPRi-mediated *lncGRS-1* knockdown in internally controlled growth assays. In both GBM SF10360 and DIPG SF8628 cells, *lncGRS-1* knockdown produced growth inhibition (Fig. 4a, b) similar to that observed in U87 cultures. Thus, CRISPRi targeting of *lncGRS-1* slows the growth of GBM cell lines and primary malignant glioma cells in culture.

Antisense oligonucleotides (ASOs) degrade complementary RNAs via a ribonuclease H-based mechanism, are effective for the knockdown of nuclear lncRNAs [42], and are currently used to treat human CNS diseases [43, 44]. To confirm the ability of ASOs to efficiently deplete gene expression, we used ASOs to knockdown *TP53* (p53) in U87 cells and observed ~ 99% knockdown efficiency (Additional file 1: Fig. S4b). We then designed locked nucleic acid ASOs against *lncGRS-1* (Additional file 1: Fig. S4c, Additional file 3: Table S2) and tested them for efficacy in patient-derived glioma cell cultures. Two different ASOs against *lncGRS-1* produced a mean knockdown of 89% in patient-derived GBM SF10360 cells and 93% in patient-derived DIPG SF8628 cells (Fig. 4c). Cell proliferation was decreased by an average of 80% across both ASOs in both SF10360 and SF8628 by 11 days post-transfection (Fig. 4d). ASO-mediated knockdown of *lncGRS-1* also decreased the proliferation of patient-derived DIPG cultures SU-DIPG 24, SU-DIPG 25 (Additional file 1: Fig. S4d,e), and GBM 43 (Additional file 1: Fig. S4f). Thus, ASOs targeting *lncGRS-1* inhibit the growth of multiple human patient-derived cultures of malignant glioma.

### ASO-mediated knockdown of *lncGRS-1* is not toxic to human astrocytes

**Fig. 5:**
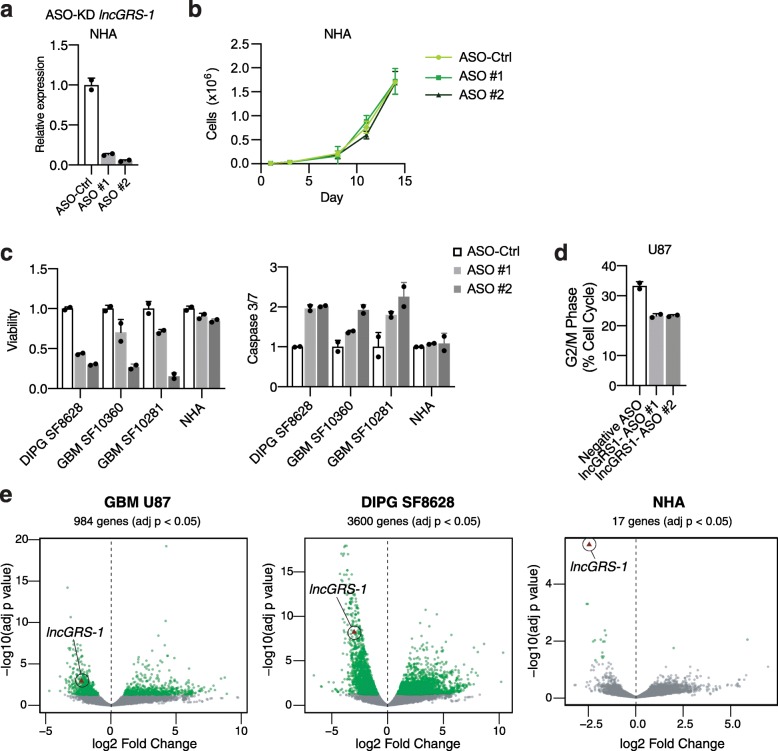
*lncGRS-1* function is glioma specific. **a** RT-qPCR of *lncGRS-1* transcript levels following ASO-mediated knockdown in NHA cells (*n* = 2 biological replicates per condition; error bars = SD). **b** Cell propagation time course in NHA cells with ASO-mediated knockdown of *lncGRS-1*. ASOs were re-transfected at day 7 (*n* = 2 biological replicates per condition; error bars = SD). **c** Left, fluorescence viability assay of malignant tumor cells and NHA cells following ASO-mediated *lncGRS-1* knockdown. Right, apoptosis assay of malignant tumor cells and NHA cells following ASO-mediated *lncGRS-1* knockdown (*n* = 2 biological replicates per condition; error bars = SD). **d** Cell cycle phase analysis following lncGRS-1 knockdown in GBM U87 using flow cytometry. **e** RNA-seq differential gene expression analysis of *lncGRS-1* knockdown in GBM U87 (left), DIPG SF8628 (middle), and NHA (right) cells using *lncGRS-1* ASO #1 and ASO #2 as biological replicates, compared to negative control ASO, 24 h following transfection (*n* = 2 biological replicate cultures per ASO condition). Green, genes adj. *p* value < 0.05. Red triangle, *lncGRS-1*

Current standard-of-care treatments for glioma are limited by toxicity to normal tissues [23]. Consistent with lncRNAs having exquisitely cell type-specific essential function [8], ASO-mediated knockdown of *lncGRS-1* did not affect the proliferation of the kidney-derived HEK293T cell line (Additional file 1: Fig. S4g). Astrocytes are the most numerous cell type of the human brain [45] and are phenotypically related to subpopulations of malignant glioma [46, 47]. We therefore next investigated the effect of *lncGRS-1* knockdown in cultures of normal human astrocytes (NHAs) [48]. Knockdown of essential gene *POLA1* (DNA polymerase alpha 1) resulted in decreased proliferation of NHA cells, in addition to U87 GBM cells (Additional file 1: Figure S4h,i). While ASO-mediated *lncGRS-1* knockdown was robustly achieved in NHA (Fig. 5a), cell proliferation was not reduced (Fig. 5b). Moreover, in NHA, measures of cell viability and apoptosis were not changed by *lncGRS-1* knockdown, whereas in patient-derived glioma cells, viability was decreased, and apoptosis was increased (Fig. 5c). Finally, CRISPRi-mediated knockdown of *lncGRS-1* with the same two sgRNAs used for knockdown in U87 cells did not result in decreased proliferation in HeLa cervical cancer cells stably expressing dCas9-KRAB (Additional file 1: Fig. S5a), despite these cells expressing *lncGRS-1* at a robust level (TPM = 4.38) among the CCLE compendium of cancer cell lines (Additional file 1: Fig. S5b) [49].

To investigate the glioma-specific phenotype of *lncGRS-1*, we performed RNA-seq analysis following ASO-mediated *lncGRS-1* knockdown. In GBM (U87), DIPG (SF8628), and NHA cells, *lncGRS-1* expression was decreased by 80–88% 24 h after ASO treatment (Fig. 5e). Consistent with the changes in viability and apoptosis observed with *lncGRS-1* knockdown, both GBM and DIPG cells exhibited transcriptome-wide differential gene expression (984 and 3600 genes adj. *p* < 0.05, respectively; Fig. 5e, Additional file 4: Table S3). Upregulated genes in U87 and SF8628 were enriched for p53 signaling and apoptosis, while downregulated genes were enriched for cell cycle and DNA damage response (Additional file 1: Fig. S5c). Despite different gene ontology terms, differentially expressed genes between U87 and SF8628 cells were positively correlated following knockdown of *lncGRS-1* (Pearson’s *R* = 0.667; Additional file 1: Fig. S5d). Consistent with these genome-wide changes, levels of *CDKN1A* (p21) increased at the level of both mRNA and protein following *lncGRS-1* knockdown in U87 cells (Additional file 1: Fig. S5e,f, Additional file 1: Fig. S6). These cells also exhibited decreased proportions of G2/M phase in population-level flow cytometry (Fig. 5d). Interestingly, while *lncGRS-1* knockdown resulted in increased p53BP1 foci in U87 cells in the absence of radiation, γH2AX foci induced by radiation were potentiated by knockdown of *lncGRS-1*, yet *lncGRS-1* knockdown in the absence of radiation did not generate additional γH2AX foci (Additional file 1: Fig. S5g,h). Remarkably, while ASOs were effective for *lncGRS-1* knockdown in NHA, such genome-wide changes to the transcriptome were not observed in these non-tumorigenic cells (Fig. 5e).

### ASOs targeting *lncGRS-1* decrease glioma tumor growth in mature brain organoids

**Fig. 6:**
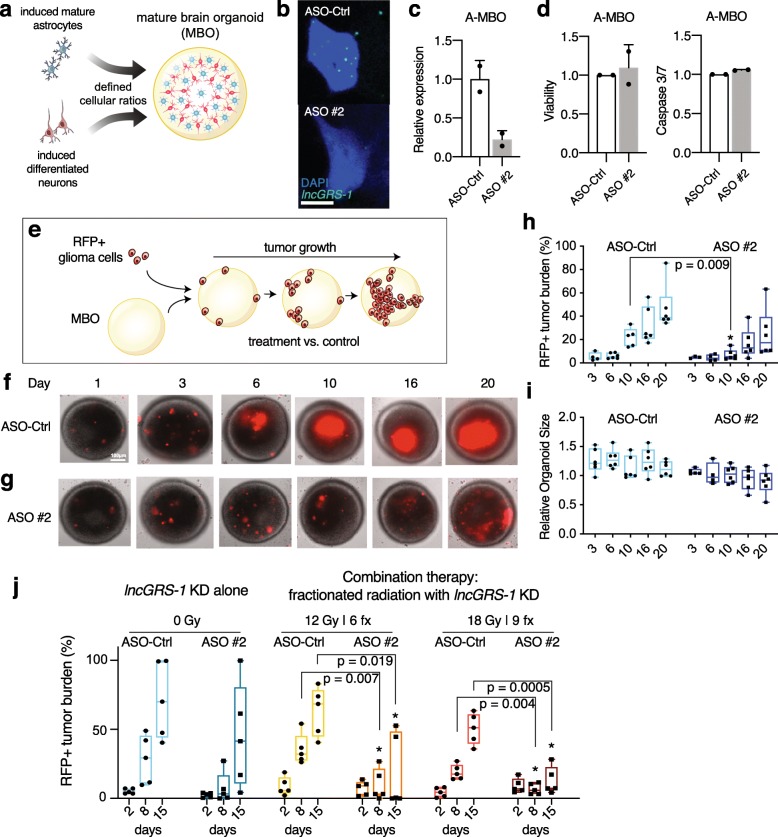
Tumor-specific, radiosensitizing function of *lncGRS-1* knockdown in mature brain organoids (MBOs). **a** Schematic of MBO assembly from induced mature astrocytes (iAstrocytes) and induced neurons (i^3^Neurons). **b** Single molecule RNA FISH of *lncGRS-1* in iAstrocyte MBO (A-MBO) cells following transfection of non-targeting ASO (top) or ASO targeting *lncGRS-1* (bottom). Scale bar = 5 μm. **c** RT-qPCR of *lncGRS-1* in A-MBOs seeded with DIPG SF8628 following ASO transfection (*n* = 2 biological replicates per condition; error bar = SD). **d** Left, fluorescence viability assay of A-MBOs following transfection of non-targeting ASO or ASO targeting *lncGRS-1*. Right, apoptosis induction assay of A-MBOs following transfection of ASOs (*n* = 2 biological replicates per condition; error bar = SD). **e** Schematic of RFP+ glioma cell seeding and subsequent RFP+ tumor growth over time. **f**, **g** Longitudinal fluorescence microscopy of A-MBOs seeded with RFP+ DIPG SF8628 cells treated with non-targeting Ctrl ASO (**f**) or ASO targeting *lncGRS-1* (**g**). **h**, **i** Quantification of RFP+ tumor burden (**h**) and organoid diameter (**i**) in longitudinal analysis of A-MBOs seeded with RFP+ DIPG SF8628 cells (*n* = 6 biological replicates per condition; two-tailed Student’s t test). **j** Quantification of RFP+ U87 GBM tumor burden in iAstrocyte and i^3^Neuron MBOs (AN-MBOs) treated with ASOs with or without radiation (*n* = 5 biological replicates per condition; two-tailed Student’s t test). Boxplots represent 1st quartile, median, and 3rd quartile with whiskers = range

While the genomic sequences of *lncGRS-1* are conserved across primates, orthologs of this lncRNA do not exist in lower vertebrates such as mice, chicken, and zebrafish (Fig. 3a, Additional file 1: Fig. S3). Therefore, evaluating the potential toxicity of *lncGRS-1* knockdown in normal postnatal brain necessitates an alternative to xenograft mouse models. Recent studies have shown that embryonic cerebral organoids can serve as 3D tissue “hosts” for the growth of human glioma cells [30]. However, such embryonic brain organoids are comprised mostly of immature, proliferative neural progenitors and contain very few astrocytes [50–52], which are a type of glia essential to neuronal viability and function [53]. We therefore generated “mature” human brain organoids (MBOs) that more closely reflect the differentiated cellular state of the postnatal human brain (Fig. 6a).

Astrocytes are the most abundant cell type of the adult human brain [53]. We generated pure populations of mature human astrocytes from human iPSCs (iAstrocytes) using a protocol that allows maturation for at least 6 months [54, 55]. Using an isogenic iPSC (WTC11) clone that carries an inducible *Neurogenin2* (*NGN2*) transgene, we also generated homogenous cultures of mature cortical neurons (i^3^Neurons) with *NGN2* induction [54, 56]. MBOs can be formed from iAstrocytes and i^3^Neurons by mixing and co-culture of these cell types in defined numbers and ratios (from a 1:1 ratio to solely iAstrocytes or i^3^Neurons) (Fig. 6a) [55].

To build upon our studies of NHAs in 2D culture, we first studied MBOs assembled from iAstrocytes (A-MBOs). Unlike astrocytes in 2D culture, iAstrocytes in MBOs become highly ramified and develop complex structures similar to those observed in human brain tissue [55]. Transfection of A-MBOs with ASOs against *lncGRS-1* was effective for its knockdown as assessed by both in situ hybridization, demonstrating depletion of *lncGRS-1* within nuclei (Fig. 6b), and RT-qPCR (Fig. 6c, Additional file 1: Fig. S7a). Similar to our results from NHAs in 2D culture (Fig. 5a–c), ASO-mediated *lncGRS-1* knockdown in A-MBOs did not reduce organoid viability or increase apoptosis (Fig. 6d).

We next used A-MBOs as the “host” for human glioma tumor growth (Fig. 6e). After DIPG SF8628 cells labeled with red fluorescent protein (RFP) were seeded to the surface of these MBOs, RFP+ tumors grew progressively larger within the organoid tissue in an invasive manner, when individually monitored using serial microscopic imaging (Fig. 6f). For treatment of a human CNS disease, ASOs are administered weekly directly into the cerebrospinal space [57]. Mimicking this dosing schedule, we added ASOs into the organoid culture media every 7 days. In each organoid, the growth of RFP+ tumors was prospectively imaged (Fig. 6f, g), and tumor burden was estimated by quantification of the RFP signal. ASOs against *lncGRS-1* reduced DIPG tumor growth as compared to non-targeting, negative control ASOs (Fig. 6h). Consistent with our results of organoid viability and apoptosis without infiltrated tumor cells (Fig. 6d), the overall size of the host brain organoid was not changed by *lncGRS-1* knockdown (Fig. 6i).

We further developed the MBO-glioma model by using MBOs assembled from both iAstrocytes and i^3^Neurons (AN-MBOs) and also including radiation therapy. ASO-mediated knockdown of *lncGRS-1* did not affect the viability of AN-MBOs (Additional file 1: Fig. S7b). Furthermore, treatment of AN-MBOs with clinically therapeutic levels of fractionated radiation (total dose up to 54 Gy), with or without *lncGRS-1* knockdown, did not affect the overall size of organoids over a period of 3 weeks (Additional file 1: Fig. S7c). Using this AN-MBO model of radiotherapy, we investigated whether *lncGRS-1* knockdown sensitizes glioma cells to the therapeutic effects of radiation. RFP-labeled U87 cells were seeded to the surface of individual AN-MBOs isolated across multiple wells of a 96-well dish, and tumors grew invasively (Additional file 1: Fig. S7d) and progressively larger when treated with negative control ASOs and no radiation when tracked using serial microscopic imaging (Additional file 1: Fig. S7e). As expected, radiation treatment alone (control ASOs with increasing doses of radiation) exhibited a trend of tumor inhibition (Fig. 6j). Notably, when radiation (18 Gy in 9 fractions, or 12 Gy in 6 fractions) was combined with ASO-mediated *lncGRS-1* knockdown, the tumor burden was significantly lower than that observed with radiation alone (Fig. 6j, Additional file 1: Fig. S7e). Thus, ASO-mediated knockdown of *lncGRS-1* sensitizes malignant glioma to the therapeutic effects of radiation in this 3D model of tumor growth.

## Discussion

In this study, we developed a CRISPRi-based pooled screening platform to discover novel non-coding therapeutic targets in human malignant glioma that can enhance the efficacy of radiation therapy. Given that CRISPRi is effective in a wide range of cancer cell types [8], our work presents a conceptual and experimental framework that can be used to rapidly interrogate new targets in multiple, clinically relevant treatment combinations (e.g., synthetic lethality with traditional chemotherapy and/or radiation, or with newer classes of targeted therapeutics). The prioritization of combinatorial therapeutic targets for preclinical development is increasingly needed, particularly for malignant glioma [58–60]. While we focused on lncRNAs as potential therapeutic targets in glioma, the overall strategy described here could be easily adapted to screen other types of non-coding genomic elements as well as coding genes.

The interaction between radiation treatment and any particular biological target can be difficult to predict, especially when the mechanism of action of the target is not fully understood. In our study of lncRNAs, of the 467 screen hits that reduced the growth of irradiated GBM cells, only 33 hits behaved as sensitizers (having effect sizes greater with fractionated radiotherapy than without radiation). Interestingly, some lncRNA hits that reduced the growth of non-irradiated GBM cells appeared to ameliorate the effect of radiation (Fig. 2c). These results highlight the importance of unbiased functional genomic methods such as CRISPRi-based pooled screening, particularly when investigating new classes of potential therapeutic targets.

Our systematic approach prioritized *lncGRS-1* as a top radiation sensitizer for malignant glioma. Knockdown of *lncGRS-1* with either CRISPRi or ASOs inhibited the growth of both adult and pediatric forms of malignant glioma, potentiating the therapeutic effects of clinically relevant doses of fractionated radiotherapy. Therefore, *lncGRS-1* shares properties both of an essential gene that is required for normal growth and also a radiation sensitizer target. However, despite being expressed in non-malignant brain cells, *lncGRS-1* knockdown did not appear to adversely affect their viability or growth. Of particular note, ASO-mediated knockdown of *lncGRS-1* in NHA—cells that proliferate as briskly as the patient-derived glioma cells—did not impair the growth of this non-malignant glial cell type, nor did *lncGRS-1* knockdown affect growth of the malignant but non-glioma cell line HeLa. While the transcriptomes of GBM and DIPG cells were broadly perturbed by *lncGRS-1* knockdown, only 17 genes were differentially expressed (with *lncGRS-1* being the most significant) in NHAs with the same ASO-mediated knockdown. The mechanisms underlying this glioma-specific function of *lncGRS-1* remain to be discovered, but upon *lncGRS-1* knockdown, we did observe activation of the p53 signaling pathway leading to upregulation of p53 effectors such as p21, correlating with a decrease in cell cycle progression. Our observations here build importantly on previous genome-scale studies that reveal essential lncRNAs as having exquisitely cell type-specific function [8, 61, 62]. Based on this emerging understanding of lncRNA biology, we speculate that lncRNAs as a class are enriched for targets that have important roles selectively in malignant cells.

Modeling human glioma in mice as transplanted xenografts is time-consuming and relatively expensive, particularly for early preclinical studies intended to screen new drugs for therapeutic efficacy and potential toxicity [25]. Furthermore, certain human therapeutic targets do not exist in animal hosts—as is the case for *lncGRS-1*—decreasing the utility of animal models for assessing potential toxicity stemming from the ablation of such targets in normal tissue. Human organoids represent a medium-throughput platform for cancer studies [63], and our studies demonstrate the utility of MBOs for simultaneously evaluating treatment efficacy and brain toxicity, including in the context of current radiotherapy treatment paradigms.

As a 3D tissue platform for the study of human glioma, MBOs offer certain characteristics that distinguish them from embryonic brain organoids and GBM-derived tumor organoids. In contrast to embryonic brain organoids that mimic early stages of fetal brain development, MBOs are assembled from cell populations that are more mature and postmitotic [54, 55]. Because embryonic brain organoids contain a large proportion of proliferative precursor cells, radiation treatment and/or other traditional chemotherapies may not be well-tolerated by such normal but immature cells. GBM-derived tumor organoids are useful for the study of drug efficacy in 3D tissues, but because they are comprised of only tumor cells, toxicity to normal cells and therefore the therapeutic index [64] cannot be assessed. Because of these differences, it is likely that each organoid platform will have important utility in different preclinical research scenarios. As a bridge between in vitro and in vivo preclinical experiments intended to prioritize drug candidates and therapeutic targets, MBOs have shown utility in our study, identifying *lncGRS-1* as a target for further preclinical development such as in vivo validation. One limitation of our MBO-glioma model is the absence of a complete tumor microenvironment, which includes microglia, stromal cells, and tumor-infiltrating lymphocytes, among other cell types [65, 66]. However, recent advances in tumor organoid derivation have demonstrated preservation of syngeneic tumor-infiltrating lymphocytes and stromal fibroblasts along with neoplastic cells using an air liquid interface [67]. Analogous strategies, in addition to the incorporation of vascular endothelial cells, may further augment the experimental utility of MBOs in future iterations.

## Conclusions

There is an unmet need for cancer therapies that potentiate the therapeutic effects of radiation therapy while minimizing toxicity [59]. Radiation therapy is one of the most common treatment modalities for all cancers, and it is nearly always indicated for patients with malignant glioma [23]. The development of drug-radiotherapy combinations has generally been pursued with low-throughput, non-systematic approaches [60]. Our genome-scale CRISPRi-based radiation modifier screen of lncRNA loci in glioma demonstrates a strategy for revealing novel therapeutic targets in this vast, largely unexplored aspect of the non-coding genome. More broadly, we anticipate that the coupling of large-scale screening efforts with target validation in MBO models will serve as a useful framework for accelerating the development of new therapeutics (and combinations) through the preclinical research pipeline.

## Methods

### Cell culture and radiation treatment

U87, NHA, and HEK293T cells were grown in DMEM with 10% FBS and antibiotics/antimycotics. DIPG SF8628, GBM SF10360, and GBM 43 were cultured in N5 (Neurobasal-A (1×), N2 (1×), B27 supplement without vitamin A (1×), l-glutamine (1×)), 5% FBS, and FGF and EGF (20 ng/mL each). SU-DIPG 24 and SU-DIPG 25 were cultured in TSM (Neurobasal-A (1×), DMEM/F12 (1×), HEPES (10 mM), sodium pyruvate (1 mM), MEM non-essential amino acids solution (0.1 mM), GlutaMax supplement (1×), antibiotic-antimycotic (1×)) with EGF (20 ng/mL), FGF-basic-154 (20 ng/mL), PDGF-AA (10 ng/mL), PDGF-BB (10 ng/mL), and heparin (2 μg/mL). Proliferation was measured using a manual hemocytometer. Cell viability was measured using the CellTiter-Blue Cell Viability Assay (Promega), and apoptosis was measured using the Apo-ONE Homogenous Caspase-3/7 Assay (Promega). Radiation was delivered using a Cesium-137 irradiator with rotating platform, with cells treated while in suspension for the CRISPRi screen and after achieving adherence on plates for validation assays.

Determination of radiation synergy was calculated as follows. The null model of additive effects was determined by multiplying the mean decrease in cell proliferation caused by radiation treatment alone by the decrease in cell proliferation following gene knockdown. Synergistic effects were identified if the observed absolute decrease in cell proliferation following combination of lncRNA knockdown and radiation was significantly greater than the predicted decrease in cell proliferation based on the additive model (two-tailed Student’s *t* test).

### CRISPRi screens

sgRNA library was derived from the CRISPRi Non-Coding Library (CRiNCL) [8], selecting sub-libraries that targeted all expressed lncRNAs in U87: Common + Cancer common + (U87, HEK293T) + U87 unique. sgRNAs were cloned into the library expression vector pCRISPRia-v2 [11, 68], and lentivirus pools were generated as previously described [8]. U87-dCas9-KRAB cells were generated previously in [69]. Lentivirus libraries were infected in duplicate cultures, cultured for 2 days following infection, puromycin (1 mg/mL) selected for 2 days, and recovered for 1 day without puromycin. Cells were then cultured for 12 days at a minimum coverage of 1000×, starting at this “T0.” For the radiation modifier screen, doses of 2 Gy radiation were given at the following days: T0, T2, T4, and T6, for a total of 8 Gy fractionated ionizing radiation. Genomic DNA was harvested from aliquots of ~ 60 M cells each from T0 and T12 and processed for sequencing as previously described [11, 68]. Data processing and hit analysis was performed as described in [8], with the exception that neighbor hits were considered to be hits whose TSS were within 1 kb of any protein coding gene TSS expressed in U87. Growth-only screen data for U87 was obtained from [8] and compared to the radiation screen data obtained in this study. For the identification of radiation sensitizers, the screen scores (defined as the average phenotype of the top three sgRNAs against a given gene multiplied by the negative log10(Mann-Whitney *U p* value) for that gene) for the radiation screen was compared to those of the growth screen for all genes targeted in both screens. Phenotype in these CRISPRi screens refer to the relative log2 enrichment of barcodes in the final timepoint divided by the enrichment of barcodes at the initial timepoint, as has been previously described [8, 11]. A screen score threshold of 5, which corresponded to an empirical false discovery rate of 0.25%, was applied to both screens, and hits with radiation scores greater than growth scores were retained. LncRNAs with evidence of expression in primary glioma cells were then identified as *lncGRS*. *Z* standardized growth and radiation phenotypes were calculated as log2 enrichment normalized by the standard deviation of negative control genes’ phenotypes. Sensitizer score was defined as the ratio of the radiation modifier screen score in irradiated cells to the growth screen score in non-irradiated cells.

sgRNA validation and internally controlled growth assays was performed as described in [8], with the addition of 4 fractions of 2 Gy radiation starting 2 days following sgRNA infection, delivered every other day. Purified populations of sgRNA-expressing cells were selected in 1 mg/mL puromycin for 3 days before assay. Internally controlled CRISPRi growth assays were performed as previously described, briefly, by partially infecting a population of cells stably expressing dCas9-KRAB with a fluorescently labeled sgRNA expression vector and tracking the sgRNA-infected population over time using flow cytometry relative to the non-infected population [8]. sgRNA protospacer sequences for individual knockdowns are listed in Table S2.

### RT-qPCR

RNA was harvested in TRIzol at 24 h following ASO transfection, or in the case of CRISPRi, 72 h following initiation of puromycin selection for sgRNA expression. RNA was purified using the Direct-zol MiniPrep or MicroPrep RNA purification kits (Zymo Research) with the on-column DNAse digestion step. cDNA was generated using the Transcriptor First Strand cDNA Synthesis Kit (Roche) and diluted 1:5 fold before proceeding to qPCR. qPCR was performed using the SYBR Green I Master Mix (Roche) on a LightCycler 480 instrument (Roche). qPCR primers are listed in Table S2.

### Subcellular fractionation

Cells were plated in a 15-cm dish and fractionated as previously described [70]. Briefly, 10 to 20 million cells were collected, washed with phosphate-buffered saline (PBS), and resuspended at 4 × 10^7^ cells/mL in buffer A (10 mM HEPES [pH 7.9], 10 mM KCl, 1.5 mM MgCl2, 0.34 M sucrose, 10% glycerol, 1 mM dithiothreitol, and protease inhibitor cocktail [Boehringer]). Triton X-100 was added (0.1% final concentration), the cells were incubated on ice for 8 min, and nuclei (fraction P1) were collected by centrifugation (5 min, 1300×*g*, 4 °C). The supernatant (fraction S1) was clarified by high-speed centrifugation (5 min, 20,000×*g*, 4 °C), and the supernatant (fraction S2) was collected. The P1 nuclei were washed once in buffer A and lysed for 30 min in buffer B (3 mM EDTA, 0.2 mM EGTA, 1 mM dithiothreitol, and protease inhibitor cocktail [Boehringer]), and insoluble chromatin (fraction P3) and soluble (fraction S3) fractions were separated by centrifugation (5 min, 1700×*g*, 4 °C). The P3 fraction was washed once with buffer B. All the fractions obtained were resuspended in TRIzol for RNA extraction, and qPCR was performed as described above.

### Single molecular FISH

ISH was performed on cell lines and MBOs using the RNAscope 2.5 HD Assay—BROWN (Advanced Cell Diagnostics). Probes targeting the *lncGRS-1* transcript were used (RNAscope Probe Hs-CTC-338 M12.4, catalog number 300031). ISH was performed following the manufacturer’s instructions.

### Western blot

Cells were washed with PBS and lysed using RIPA buffer supplemented with HALT protease inhibitor (78429; ThermoFisher Scientific). The lysate was mixed (1:1) with 2× NuPAGE LDS Sample Buffer and ran on NuPAGE 10% Bis-Tris gel using NuPAGE MOPS SDS Running Buffer. Proteins were transferred from gel to Amersham Hybond PVDF membrane using NuPAGE Transfer Buffer with 10% methanol. The membrane was blocked for 1 h in Odyssey Blocking Buffer and incubated overnight at 4 °C with primary antibodies—p21 (2947), 1:1000, and GAPDH_14C10(2118),1:1000, both from Cell Signaling Technologies. Following 3× 10 min washes with PBS+ 0.1% Tween, the membrane was probed by the IRDye 800CW Goat anti-Rabbit IgG (926-32211). Images were captured using LiCOR Odyssey Infrared Imaging Systems and quantified using ImageJ. Western blot intensities were normalized by their respective loading controls and then normalized again to the mean of the negative control conditions.

### Immunohistochemistry for P53BP1 and γH2AX

Cells were plated in 8-well chambered slides (Thermo Scientific Nunc 154526) at a density of ~ 10,000 cells/cm^2^ and cultured overnight. Each chamber was transfected with either control ASO or *lncGRS-1* ASO and then irradiated at 2 Gy. Cells were fixed 6 h following radiation with 4% paraformaldehyde for 15 min at room temperature. They were further incubated in permeabilization reagent (0.1% Triton-X100 in TBS) for 15 min at room temperature. Cells were blocked in blocking solution (10% Normal Donkey Serum in TBS) for 1.5 h in a humidified chamber. Cells were incubated in primary antibody solution anti-rabbit Phospho-53BP1 (Ser1778, Cell Signaling Technologies) at a 1:100 concentration and anti-rabbit γH2AX (Ser139, Cell Signaling Technologies) at a 1:400 concentration. Plates were incubated overnight at 4 °C. Cells were washed in PBST, and secondary antibody and DAPI were added (Alexa 488 for P53BP1 and Alexa 594 for γH2AX) at a 1:1000 concentration. Plates were incubated for 2 h at room temperature in a humidified chamber away from light. Slides were washed and mounted with Aqua-PolyMount. Slides were left to dry in the dark at room temperature for 3 h. Images were acquired at room temperature on an SP3 Leica Confocal microscope using × 63 oil objective and processed using ImageJ.

### Flow cytometry for cell cycle analysis

Cells were transfected with ASOs as described below. After 72 h, cells were pulsed with 33 μM bromodeoxyuridine (BrdU) for 20 min, and afterwards fixed in 70% ethanol. Cells were then stained with primary anti-BrdU antibody (Clone B44; BD Biosciences) for 1 h, followed by 1 h incubation with Alexa Fluor 488 anti-mouse IgG (Invitrogen). DNA was counterstained using 0.1 mg/mL propidium iodide supplemented with RNase for 1 h at 37 °C. Analysis was performed on a FACSCalibur using CellQuest (BD). Quantification and analysis of cell cycle profiles were performed using FlowJo (Tree Star, Inc).

### Antisense oligonucleotides

Locked nucleic acid antisense oligonucleotides were designed using the Qiagen custom LNA oligonucleotides designer. ASOs were transfected at a final concentration of 50 nM using the Lipofectamine RNAiMAX Reagent using the manufacturer’s instructions (Thermo). Three ASOs were tested, and the top two based on knockdown efficiency were used for subsequent studies. For organoid transfection experiments, the ASO with the highest knockdown efficiency was used. For ASO penetration control studies, 5′-FAM-labeled ASOs of negative control ASO A were used (Qiagen). ASO target sequences are listed in Table S2.

### Nanopore direct RNA sequencing

Total RNA was isolated from U87 human glioblastoma cells using TRI Reagent Solution (ThermoFisher), followed by bead-based poly(A) selection. Approximately 750 ng of poly(A) RNA was used for dT adapter ligation, followed by reverse transcription, and additional ligation of motor adapter prior to loading onto the Oxford Nanopore Technologies (ONT) PromethION for sequencing. The ionic current trace for each poly(A) RNA strand was base called using the ONT Guppy algorithm.

### RNA-seq sample preparation and data analysis

For U87 radiation assays, RNA was harvested using TRIzol 48 h following radiation treatment and purified using the Direct-zol MiniPrep RNA purification kits (Zymo Research) with the on-column DNAse digestion step. For ASO assays followed by RNA-seq, RNA was harvested at 24 h following ASO transfection. RNA integrity was confirmed using the Agilent Bioanalyzer. RNA-seq libraries were generated using TruSeq Stranded mRNA kit according to the manufacturer’s protocol (Illumina). cDNA was validated using the Agilent Bioanalyzer, Qubit 2.0 Fluorometer (Life Technologies), and ddPCR (Bio-Rad). Cluster generation and sequencing was performed on a HiSeq 2500, using the paired end 100 read protocol.

Reads were aligned to the human genome (GRCh38) using the spliced read aligner HISAT2 v2.0.3 [71] against an index containing SNP and transcript information (*genome_snp_tran*). Quantification of Ensembl build 75 genes was carried out with featureCounts [72] using only uniquely mapped reads. Differential expression analysis was performed using DESeq2 [73] using the Wald test with an adjusted (multiple hypothesis corrected) *p* value threshold of 0.05 as threshold for differential expression. Complete linkage hierarchical clustering was performed using 1 – Pearson’s correlation coefficients as the distance matrix, using only differentially expressed genes or differentially expressed lncRNAs. Gene ontology terms were obtained using Enrichr [74], taking gene names from the clusters of all upregulated or downregulated genes. Analysis was performed using R version 3.6.

### Mature brain organoids and tumor co-cultures

#### Generation of iAstrocytes

WTC11 human iPSCs were directed towards a cortical astrocyte fate as previously described [54, 55]. Briefly, iPSCs were dissociated and reformed as embryoid bodies; dual SMAD inhibition was used to initiate neural induction using SB431542 and DMH1 (2 μM each) in defined media composed of DMEM F12, GlutaMax, sodium bicarbonate, sodium pyruvate, and N2 and B27 supplements. Once neuroepithelial cells were isolated, cultures were maintained in suspension for ~ 180 days using defined media composition detailed above plus EGF and FGFb (10 ng/mL each) to drive proliferation and maturation into cortical astrocytes.

#### Generation of i^3^Neurons

WTC11 human iPSCs containing a transgenic doxycycline inducible cassette of *NEUROG2* were induced into neurons as previously described [75]. Briefly, iPSCs were treated with doxycycline (2 μg/mL) in a defined media composed of DMEM F12, N2 supplement, non-essential amino acids, and GlutaMax for 3 days. Populations were characterized as postmitotic and expressing MAP 2 and βIII-Tubulin to validate neuronal induction efficiency.

#### Generation of MBO cultures

Combined iAstrocyte and i^3^Neuron mature organoid (AN-MBO) cultures were generated by combining iPSC-derived iAstrocytes and iPSC-derived i^3^Neuron at a ratio of 1:1 (iAstrocytes to i^3^Neurons), unless otherwise specified, in single cell suspension. Both iAstrocytes and i^3^Neurons were isogenic and derived from WTC11 human iPSCs. iAstrocyte mature organoids (A-MBO) were generated with iAstrocytes alone after 6–8 months generation time. Organoid spheres were generated by introducing 10,000–20,000 composite cells onto spheroid microplates (Corning). MBO cultures were prepared by combining desired cells types in single cell suspension and aliquoting the specified concentration across multiple wells of spheroid microplates (Corning). Microplates were then centrifuged at 300*g* for 3 min. Organoids were allowed to coalesce for ~ 48 h prior to the initiation of tumor cell seeding. Organoid cultures were maintained in AM0 media—DMEM/F12, N2 supplement, B27 supplement, GlutaMax, antibiotic-antimycotic (Gibco) to 1× the manufacturer’s recommended final concentrations, and heparin (2 μg/mL, Sigma).

#### DIPG cell seeding and time course

DIPG SF8628 cells labeled with lentiviral red fluorescent protein (RFP) were added as single cell suspension directly into 96-well plates, with each well containing a single pre-formed A-MBO, at a ratio of 1:5 (tumor cell to non-tumor cell). Tumor cells were seeded by pipetting directly into the culture media, and therefore, only a small proportion of tumor cells invaded each organoid after seeding (presumably only those cells that landed directly on top of the organoid). Twenty-four hours following the seeding of DIPG cells onto A-MBOs, co-cultures were transfected with ASO (50 nM) and repeated once every 7 days. Co-cultures were maintained in AM0 with EGF and FGFb (20 ng/mL each). Growth-arrested DIPG A-MBO co-cultures used for radiation dose testing were maintained in AM0 without growth factors. Phase contrast and fluorescence images were obtained by focusing on a central *Z*-plane through the center of each organoid using a Leica DMI4000 B fluorescence microscope.

#### U87 cell seeding and time course

AN-MBOs comprised of 1:1 (iAstrocytes to i^3^Neurons) at 10,000 cells of each type were prepared before tumor cell seeding. AN-MBOs were allowed to mature for 2–3 weeks in AM0. GBM U87 cells labeled with lentiviral RFP (3500 cells per co-culture) were added as single cell suspension directly into 96-well plates, with each well containing a single pre-formed AN-MBO. Twenty-four hours following the seeding of GBM U87 cells onto AN-MBOs, co-cultures were transfected with ASO (50 nM) and repeated once every 7 days. Co-cultures were maintained in AM0 without growth factors. Phase contrast and fluorescence images were obtained by focusing on a central *Z*-plane through the center of each organoid using a Leica DMI4000 B fluorescence microscope.

#### Tumor burden quantification

Two-dimensional fluorescence images acquired through the central focal plane of each organoid sphere were analyzed using ImageJ (Version 1.51 m9, NIH). The two-dimensional region of interest encompassing the MBO with tumor co-culture was calculated by manual selection of the sphere. The two-dimensional size of the RFP+ tumor infiltrate within the interior of each organoid was quantified by thresholding the fluorescence intensity to exclude non-tumor infiltrated regions and tumor-free organoids and excluding any RFP+ regions that fall outside the spherical boundaries of the total organoid co-culture surface area. Thresholding was performed in an unbiased manner with the same color intensity applied across replicates and timepoints.

#### Organoid preparation for confocal imaging

MBOs were fixed using 4% paraformaldehyde and incubated at 4 °C for 60 min. Organoids were then washed three times with PBS at room temperature, allowing 5 min incubation per wash. Organoids were then incubated for 24 h at 4 °C in PBS with 30% (wt/vol) sucrose. Organoids were embedded in disposable base molds (Fisherbrand #22363552) using embedding solution (1:1, OCT:30% sucrose solution). Embedded organoids were frozen and sliced using a cryotome producing 15 μm sections and mounted on microscopy slides. Imaging was performed using a Leica TCS SP5 X confocal microscope and analyzed in ImageJ.

## Supplementary information

**Additional file 1: Figure S1.** (a) Transcriptomic analysis of radiation response. RNA-seq analysis of differentially expressed genes following single dose radiation of U87 cells, along with significant gene ontology terms for upregulated and downregulated genes (*n* = 2-3 biological replicates per condition). (b) Log2 fold change of lncGRS-1 in U87 cells following radiation, averaged across all replicates from the same condition. **Figure S2.** Properties of the CRISPRi radiation modifier screen. **(a)** Proportion of two replicates of the screen population that are expressing sgRNA (BFP positive). Puromycin selection time period highlighted in yellow. Radiation doses indicated by arrows. **(b)** Z standardized growth (no radiation) and radiation phenotypes for *PVT1* in CRISPRi screens. **(c)** Comparison of z standardized radiation phenotypes (x-axis) and log2 fold change of targeted lncRNA expression from RNA-seq (y-axis) analysis. Z standardized phenotypes were calculated as log2 enrichment normalized by the standard deviation of negative control genes’ phenotypes. *lncGRS-1* to *lncGRS-9* are labeled by their NCBI gene names. **Figure S3.** Nanopore direct RNA-seq of spliced reads aligned to the lncGRS-1 through -9 loci in U87 cells, with GENCODE v29 transcript models, Ensembl H3K27Ac layered track, and multiz alignment for conservation (from top to bottom in each subpanel). **Figure S4.** ASO knockdown of *lncGRS-1* demonstrating glioma specific phenotype. **(a)** Single molecule RNA FISH of lncGRS-1 in DIPG SF8628 cells following transfection of non-targeting ASO (top) or ASO targeting lncGRS-1 (bottom). Scale bar = 5 μm. **(b)** RT-qPCR of TP53 (p53) transcript levels following ASO knockdown of TP53 in U87 cells. **(c)** lncGRS-1 locus with locations of sgRNA, ASO, and qPCR primer targets. **(d-g)** RT-qPCR of lncGRS-1 transcript levels (left) and cell propagation assay (right) following ASO knockdown of lncGRS-1 in SU-DIPG 24 **(d)**, SU-DIPG 25 **(e),** GBM 43 **(f)**, and HEK293T cells **(g). (h)** RT-qPCR of POLA1 transcript levels (left) and cell proliferation assay (right) following ASO knockdown of NHA cells (at day 7) or in **(i)** U87 cells (at day 3). n = 2 - 3 biological replicates per condition in all experiments indicated; error bar = S.D. **Figure S5. (a)** Cell propagation assay of purified populations of HeLa cells with lncGRS-1 CRISPRi knockdown. **(b)** Expression values (log2 (TPM + 1)) of lncGRS-1 across cell lines in the CCLE atlas, grouped by disease of origin or tissue type. **(c)** Top 5 gene ontology terms for upregulated (top) and downregulated (bottom) differentially expressed genes with adj. p val < 0.05, in GBM U87 (left) and DIPG SF8628 (right) 24 hours following lncGRS-1 ASO-mediated knockdown. **(d)** Scatter plot of genes differentially expressed in either U87 or SF8628 cell lines demonstrating positive correlation in expression changes following lncGRS-1 knockdown. **(e)** RNA-seq expression values and **(f)** western blot of protein levels for CDKN1A (p21) with quantification (right) in U87 cells following lncGRS-1 knockdown. **(g)** Immunohistochemistry of p53BP1 and (h) γH2AX nuclear foci in nuclei of U87 cells following lncGRS-1 knockdown with or without 2 Gy radiation. Scale bar = 5 μm. n = range of 225 to 440 nuclei per replicate across 2 biological replicates per condition. **Figure S6.** Full size western blot with additional replicate, corresponding to Figure S5f. **Figure S7.** Radiosensitization of glioma cells in MBO hosts. **(a)** Quantification of single molecule RNA FISH of lncGRS-1 in iAstrocyte MBO (A-MBO) nuclei following transfection of non-targeting ASO or ASO targeting lncGRS-1. *n* = 69 and 98 A-MBO nuclei quantified in ASO-Ctrl and ASO #2 conditions, respectively, across 2 independent experiments for each biological condition. **(b)** Left, fluorescence viability assay of combined (1:1 ratio) iAstrocyte and i3Neuron organoids (AN-MBO) following transfection of non-targeting ASO or ASO targeting *lncGRS-1*. Right, apoptosis induction assay of AN-MBOs following transfection of non-targeting ASO or ASO targeting *lncGRS-1.* (*n* = 3 biological replicates per condition; error bar = S.D.). **(c)** Fold change in AN-MBO size between day 2 and day 21 of co-culture with growth arrested DIPG SF8628 cells, with negative control or *lncGRS-1* ASO, at various doses of fractionated radiation. (*n* = 5 biological replicates per condition; boxplot represents 1st quartile, median, and 3rd quartile with whiskers = range). **(d)** Confocal microscopy of AN-MBO 20 days following seeding of RFP+ U87 glioma cells. Nuclei are counterstained with DAPI (blue). Scale bar = 100 μm. **(e)** Longitudinal fluorescence microscopy of individual AN-MBOs seeded with RFP+ U87 cells. Cultures were treated with non-targeting ASO (Ctrl) or ASO targeting *lncGRS-1* combined with 0 Gy, 12 Gy, or 18 Gy of fractionated radiation.

**Additional file 2: Table S1.** CRISPRi radiation screen results using sgRNAs from the CRISPRi Non-Coding Library.

**Additional file 3: Table S2.** CRISPRi sgRNA protospacer sequences used for individual knockdown, qPCR primers used, and ASO targeting sequences.

**Additional file 4: Table S3.** DESeq2 output of differentially expressed genes following ASO knockdown of *lncGRS-1* compared to negative control ASO in GBM U87, DIPG SF8628, and NHA cells.

**Additional file 5:** Review history.

## Data Availability

Raw data for the CRISPRi screen (Fig. 1a–c) are detailed in Table S1. Raw data for the RNA-seq differential expression analysis in Fig. 3d are in Table S3. RNA-seq reads, including long Nanopore reads, are deposited on SRA, accession PRJNA609239 [76]. Cells culture reagents are available by request.
