# Supplementary material for: CRISPRi-based radiation modifier screen identifies long non-coding RNA therapeutic targets in glioma
Source: Genome Biol. 2020 Mar 31;21:83. doi: 10.1186/s13059-020-01995-4 (PMC7110660; doi:10.1186/s13059-020-01995-4)
Supplement: Supplementary file 1 — Additional file 1: Figure S1. (a) Transcriptomic analysis of radiation response. RNA-seq analysis of differentially expressed genes following single dose radiation of U87 cells, along with significant gene ontology terms for upregulated and downregulated genes (n = 2-3 biological replicates per condition). (b) Log2 fold change of lncGRS-1 in U87 cells following radiation, averaged across all replicates from the same condition. Figure S2. Properties of the CRISPRi radiation modifier screen. (a) Proportion of two replicates of the screen population that are expressing sgRNA (BFP positive). Puromycin selection time period highlighted in yellow. Radiation doses indicated by arrows. (b) Z standardized growth (no radiation) and radiation phenotypes for PVT1 in CRISPRi screens. (c) Comparison of z standardized radiation phenotypes (x-axis) and log2 fold change of targeted lncRNA expression from RNA-seq (y-axis) analysis. Z standardized phenotypes were calculated as log2 enrichment normalized by the standard deviation of negative control genes’ phenotypes. lncGRS-1 to lncGRS-9 are labeled by their NCBI gene names. Figure S3. Nanopore direct RNA-seq of spliced reads aligned to the lncGRS-1 through -9 loci in U87 cells, with GENCODE v29 transcript models, Ensembl H3K27Ac layered track, and multiz alignment for conservation (from top to bottom in each subpanel). Figure S4. ASO knockdown of lncGRS-1 demonstrating glioma specific phenotype. (a) Single molecule RNA FISH of lncGRS-1 in DIPG SF8628 cells following transfection of non-targeting ASO (top) or ASO targeting lncGRS-1 (bottom). Scale bar = 5 μm. (b) RT-qPCR of TP53 (p53) transcript levels following ASO knockdown of TP53 in U87 cells. (c) lncGRS-1 locus with locations of sgRNA, ASO, and qPCR primer targets. (d-g) RT-qPCR of lncGRS-1 transcript levels (left) and cell propagation assay (right) following ASO knockdown of lncGRS-1 in SU-DIPG 24 (d), SU-DIPG 25 (e), GBM 43 (f), and HEK293T cells (g). (h) RT-qPCR of POLA1 trans [file 13059_2020_1995_MOESM1_ESM.pdf]

Figure S1

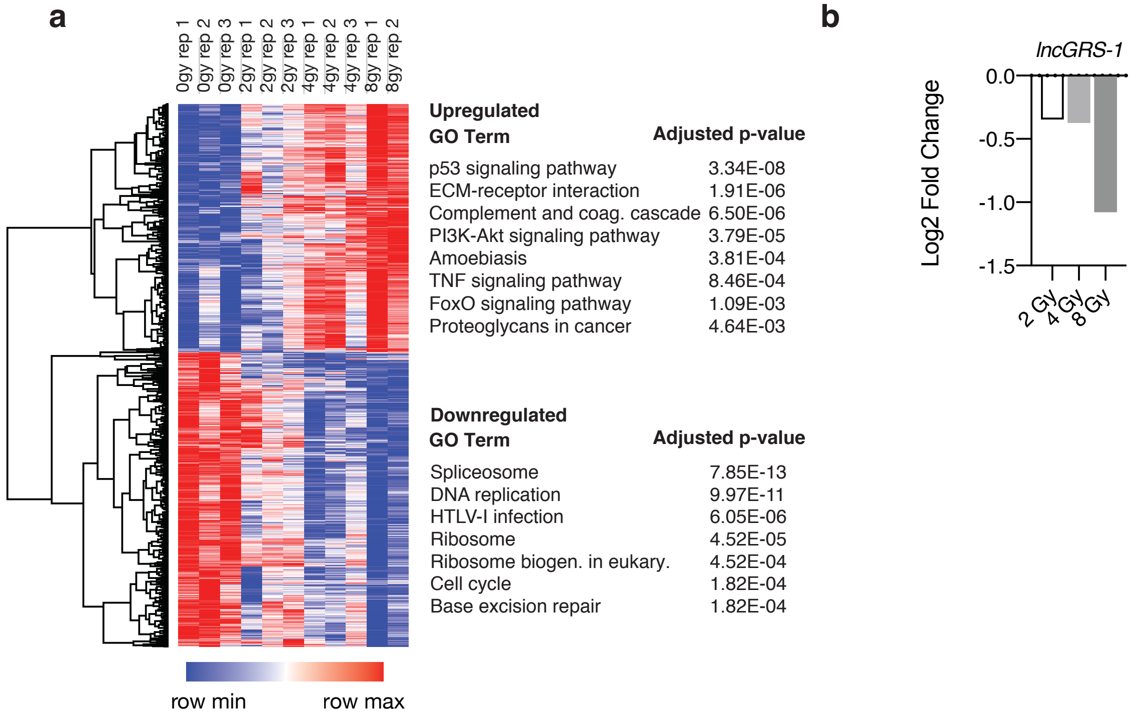

Figure S1. (a) Transcriptomic analysis of radiation response. RNA-seq analysis of differentially expressed genes following single dose radiation of U87 cells, along with significant gene ontology terms for upregulated and downregulated genes (n = 2-3 biological replicates per condition). (b) Log2 fold change of lncGRS-1 in U87 cells following radiation, averaged across all replicates from the same condition.

Figure S2

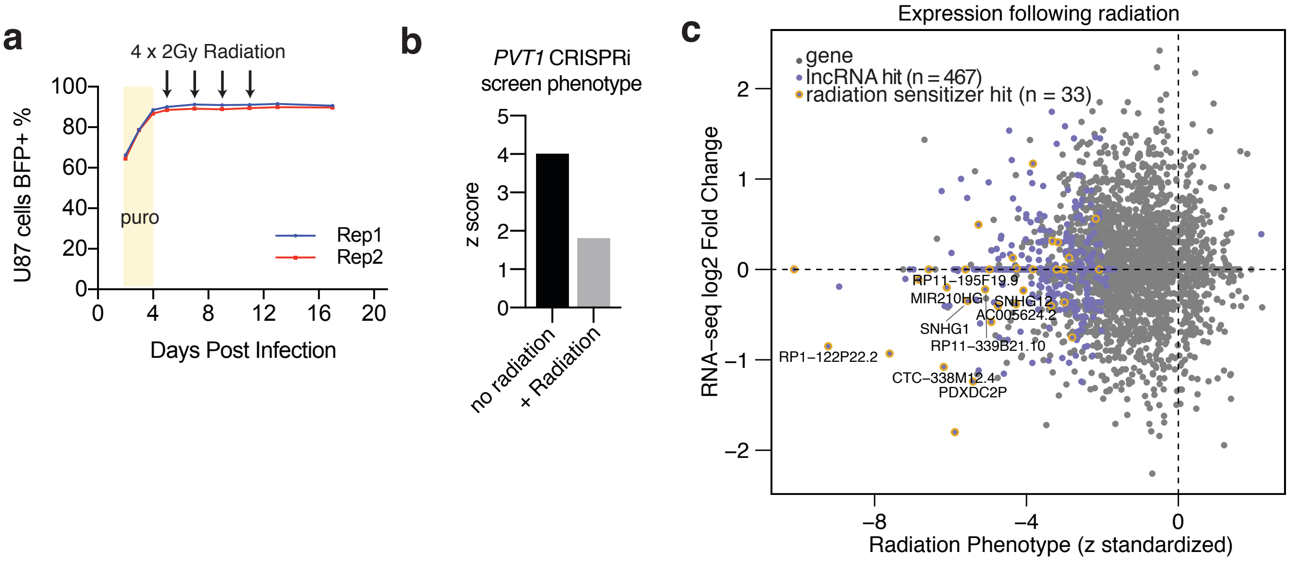

Figure S2. Properties of the CRISPRi radiation modifier screen.

(a) Proportion of two replicates of the screen population that are expressing sgRNA (BFP positive). Puromycin selection time period highlighted in yellow. Radiation doses indicated by arrows.

(b) Z standardized growth (no radiation) and radiation phenotypes for *PVT1* in CRISPRi screens.

(c) Comparison of z standardized radiation phenotypes (x-axis) and log2 fold change of targeted lncRNA expression from RNA-seq (y-axis) analysis. Z standardized phenotypes were calculated as log2 enrichment normalized by the standard deviation of negative control genes' phenotypes. *lncGRS-1* to *lncGRS-9* are labeled by their NCBI gene names.

Figure S3

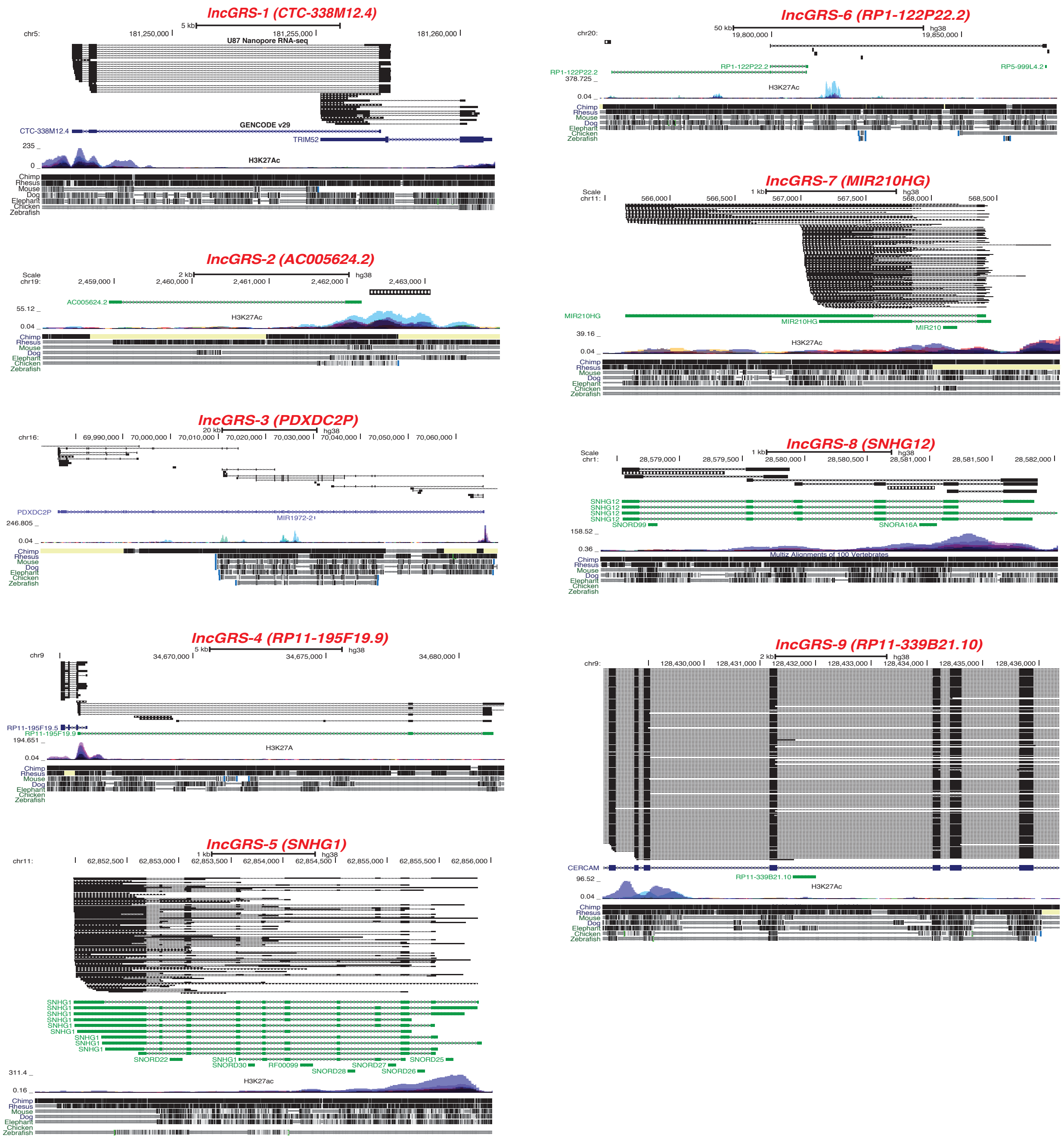

Figure S3. Nanopore direct RNA-seq of spliced reads aligned to the lncGRS-1 through -9 loci in U87 cells, with GENCODE v29 transcript models, Ensembl H3K27Ac layered track, and multiz alignment for conservation (from top to bottom in each subpanel).

Figure S4

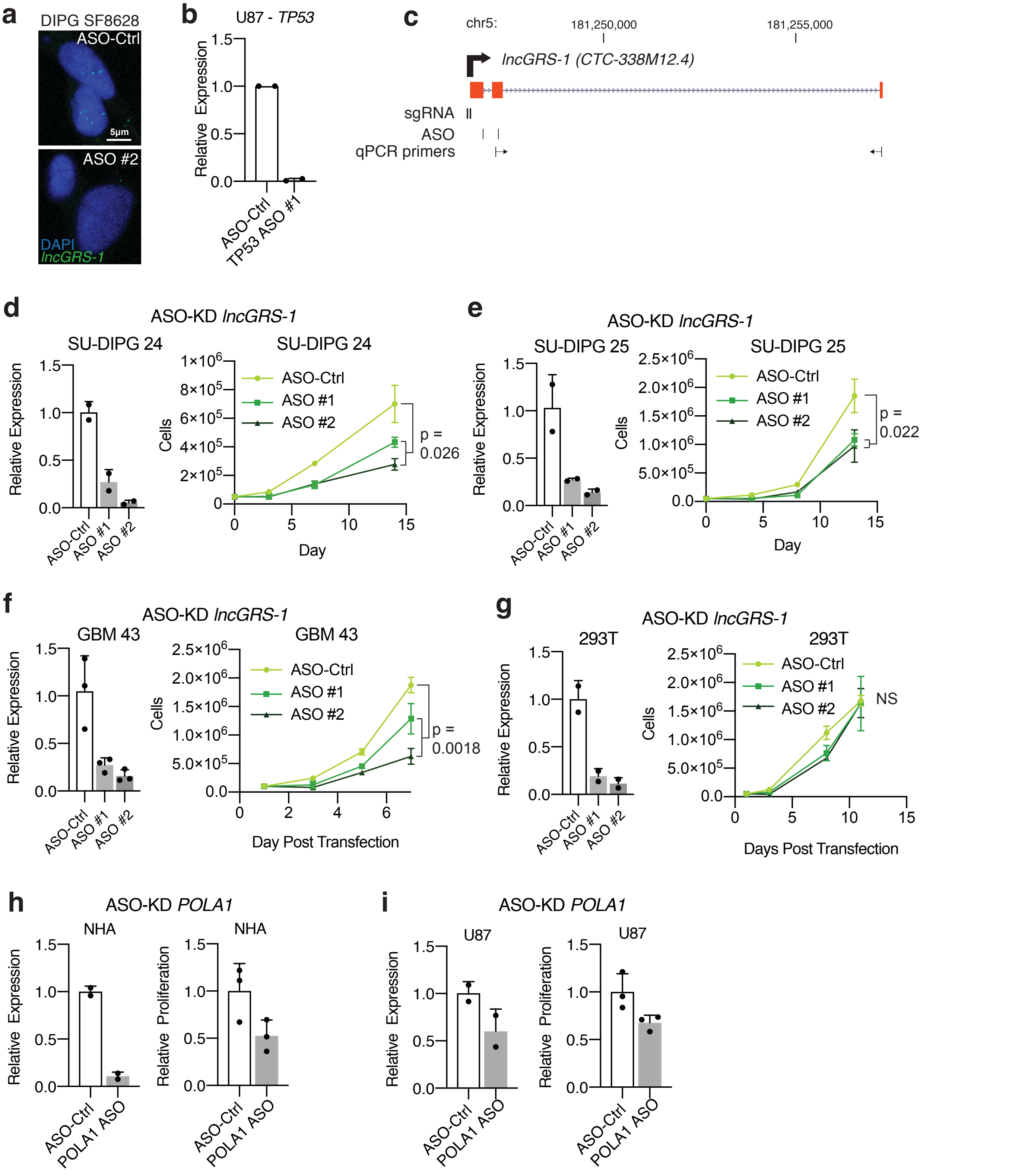

Figure S4. ASO knockdown of *lncGRS-1* demonstrating glioma specific phenotype.

(a) Single molecule RNA FISH of lncGRS-1 in DIPG SF8628 cells following transfection of non-targeting ASO (top) or ASO targeting lncGRS-1 (bottom). Scale bar = 5 μm. **(b)** RT-qPCR of TP53 (p53) transcript levels following ASO knockdown of TP53 in U87 cells. **(c)** lncGRS-1 locus with locations of sgRNA, ASO, and qPCR primer targets. **(d-g)** RT-qPCR of lncGRS-1 transcript levels (left) and cell propagation assay (right) following ASO knockdown of lncGRS-1 in SU-DIPG 24 **(d)**, SU-DIPG 25 **(e),** GBM 43 **(f)**, and HEK293T cells **(g).** **(h)** RT-qPCR of POLA1 transcript levels (left) and cell proliferation assay (right) following ASO knockdown of NHA cells (at day 7) or in **(i)** U87 cells (at day 3). n = 2 - 3 biological replicates per condition in all experiments indicated; error bar = S.D.

Figure S5

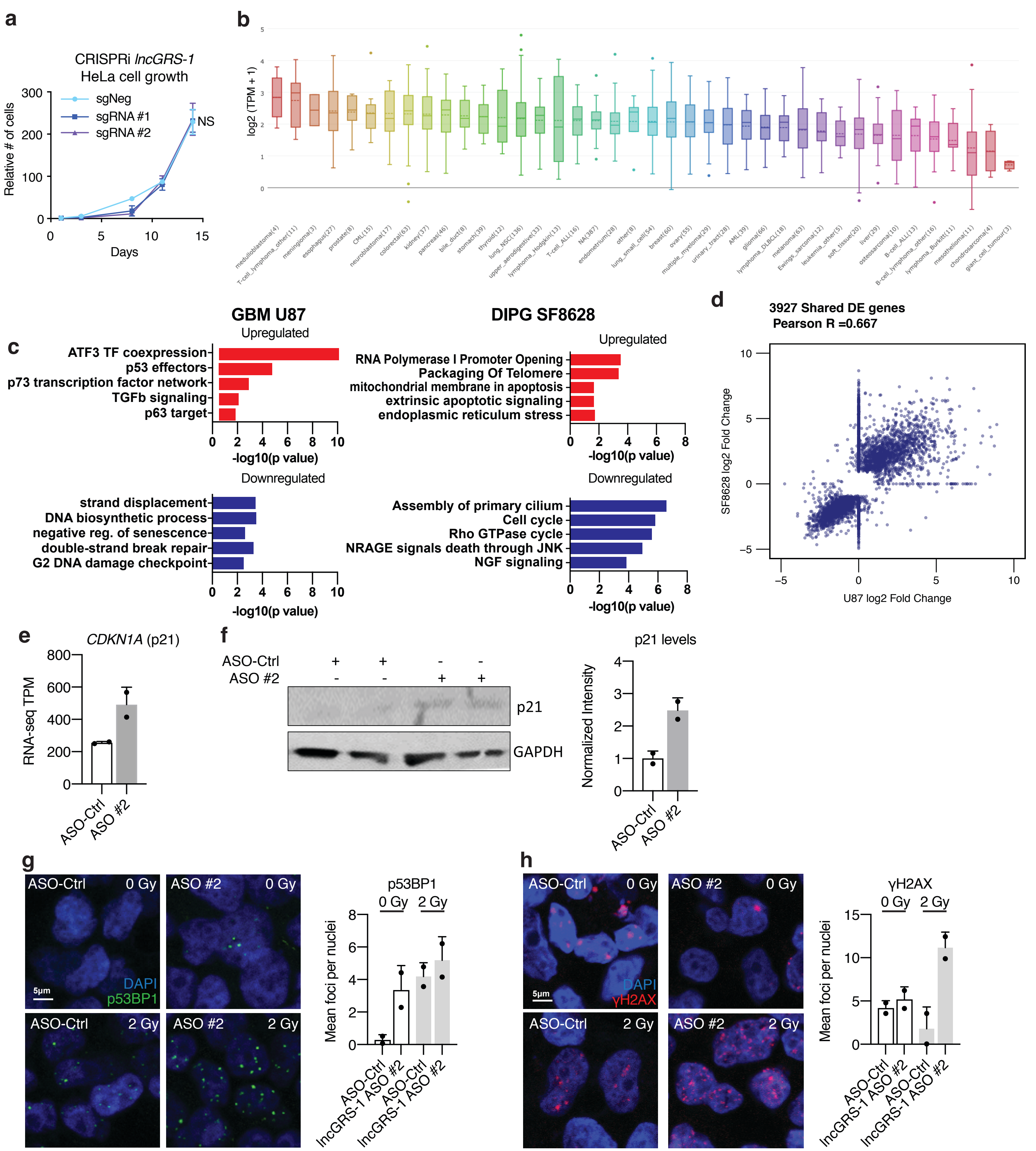

Figure S5. **(a)** Cell propagation assay of purified populations of HeLa cells with lncGRS-1 CRISPRi knockdown. **(b)** Expression values (log2 (TPM + 1)) of lncGRS-1 across cell lines in the CCLE atlas, grouped by disease of origin or tissue type. **(c)**Top 5 gene ontology terms for upregulated (top) and downregulated (bottom) differentially expressed genes with adj. p val < 0.05, in GBM U87 (left) and DIPG SF8628 (right) 24 hours following lncGRS-1 ASO-mediated knockdown. **(d)** Scatter plot of genes differentially expressed in either U87 or SF8628 cell lines demonstrating positive correlation in expression changes following lncGRS-1 knockdown. **(e)** RNA-seq expression values and **(f)** western blot of protein levels for CDKN1A (p21) with quantification (right) in U87 cells following lncGRS-1 knockdown. **(g)** Immunohistochemistry of p53BP1 and (h) γH2AX nuclear foci in nuclei of U87 cells following lncGRS-1 knockdown with or without 2 Gy radiation. Scale bar = 5 μm. n = range of 225 to 440 nuclei per replicate across 2 biological replicates per condition.

Figure S6

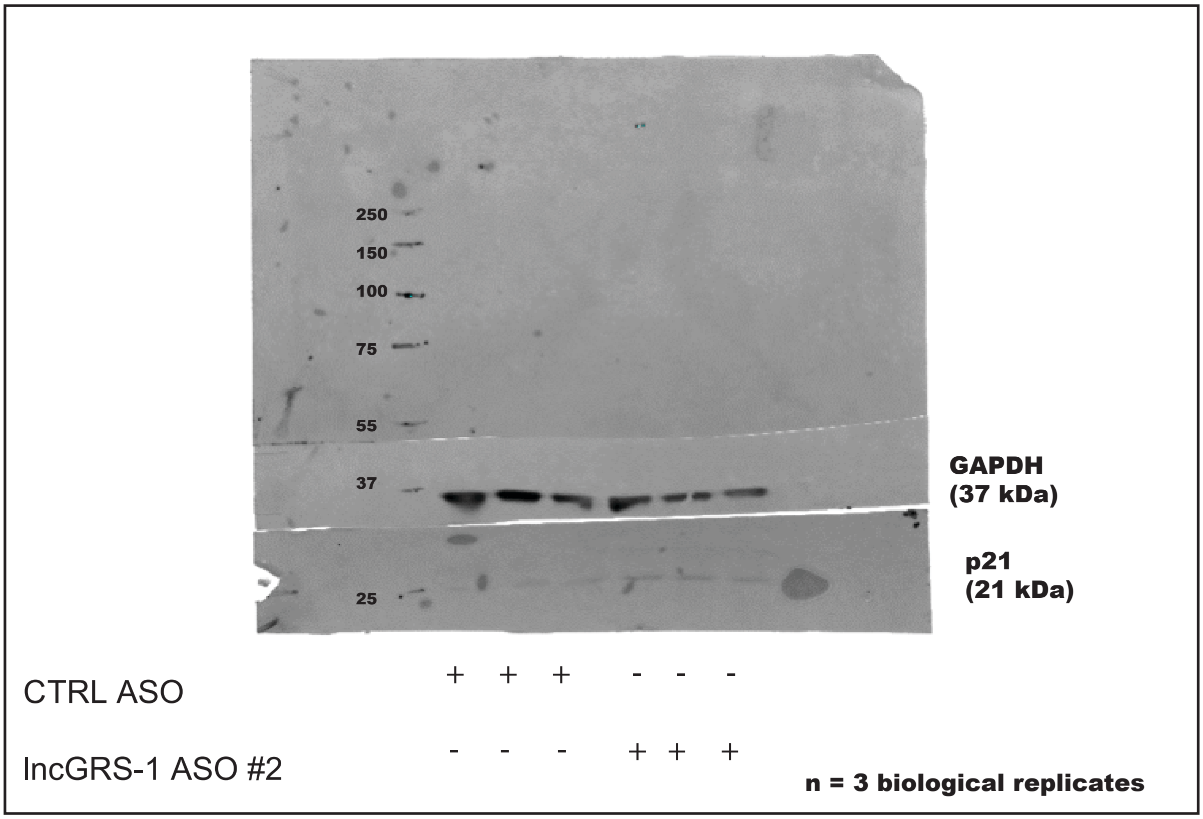

Figure S6. Full size western blot with additional replicate, corresponding to Figure S5f

Figure S7

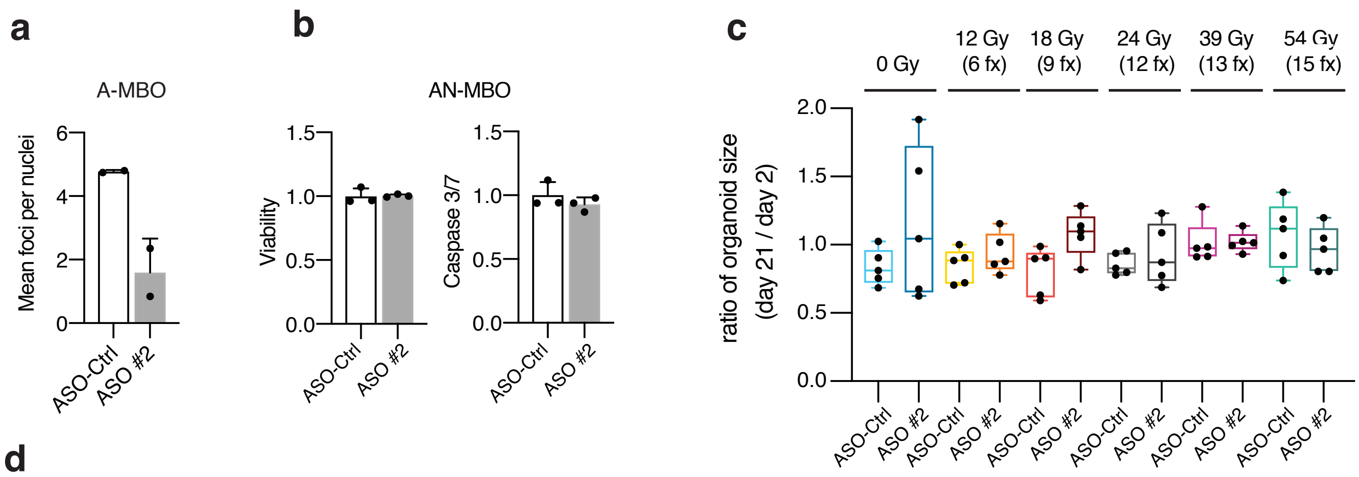

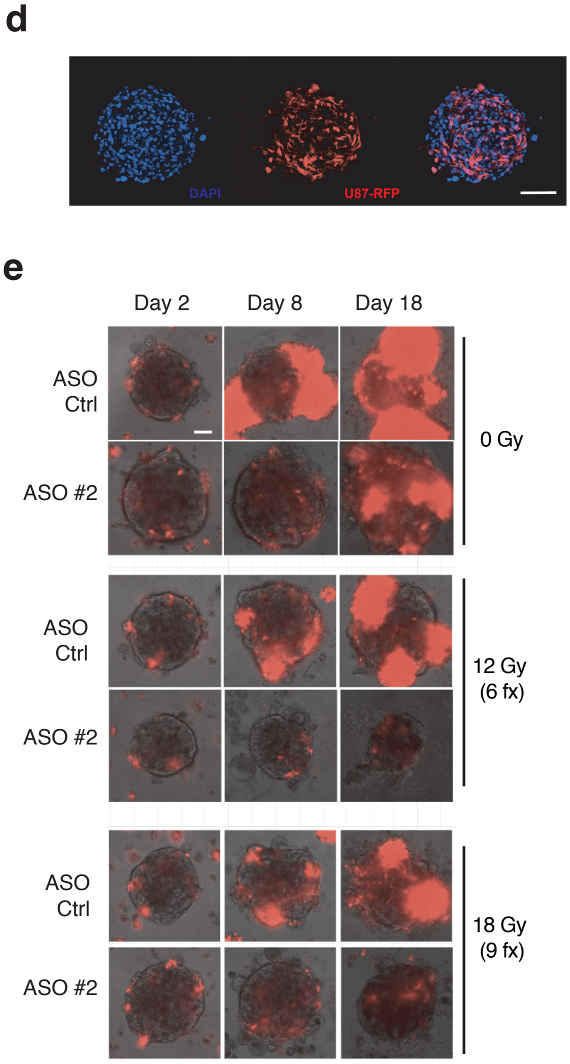

Figure S7. Radiosensitization of glioma cells in MBO hosts.

(a) Quantification of single molecule RNA FISH of lncGRS-1 in iAstrocyte MBO (A-MBO) nuclei following transfection of non-targeting ASO or ASO targeting lncGRS-1. n = 69 and 98 A-MBO nuclei quantified in ASO-Ctrl and ASO #2 conditions, respectively, across 2 independent experiments for each biological condition.

(b) Left, fluorescence viability assay of combined (1:1 ratio) iAstrocyte and i3Neuron organoids (AN-MBO) following transfection of non-targeting ASO or ASO targeting *lncGRS-1*. Right, apoptosis induction assay of AN-MBOs following transfection of non-targeting ASO or ASO targeting *lncGRS-1*. (n = 3 biological replicates per condition; error bar = S.D.).

(c) Fold change in AN-MBO size between day 2 and day 21 of co-culture with growth arrested DIPG SF8628 cells, with negative control or *lncGRS-1* ASO, at various doses of fractionated radiation. (n = 5 biological replicates per condition; boxplot represents 1st quartile, median, and 3rd quartile with whiskers = range).

(d) Confocal microscopy of AN-MBO 20 days following seeding of RFP+ U87 glioma cells. Nuclei are counterstained with DAPI (blue). Scale bar = 100 μm.

(e) Longitudinal fluorescence microscopy of individual AN-MBOs seeded with RFP+ U87 cells. Cultures were treated with non-targeting ASO (Ctrl) or ASO targeting *lncGRS-1* combined with 0 Gy, 12 Gy, or 18 Gy of fractionated radiation.
