# Supplementary material for: CRISPRi-based radiation modifier screen identifies long non-coding RNA therapeutic targets in glioma
Source: Genome Biol. 2020 Mar 31;21:83. doi: 10.1186/s13059-020-01995-4 (PMC7110660; doi:10.1186/s13059-020-01995-4)
Supplement: Supplementary file 5 — Additional file 5: Review history. [file 13059_2020_1995_MOESM5_ESM.docx]

Review History

**First round of review**

**Reviewer 1**

**Are you able to assess all statistics in the manuscript, including the appropriateness of statistical tests used?**

Yes, and I have assessed the statistics in my report.

**Comments to author:**

Using Genome-wide CRISPRi screening, this elegant study identifies the lncRNA lncGRS-1 as a glioma-specific therapeutic target and establishes an approach to rapidly identify novel therapeutic targets in the vast non-coding genome to enhance the radiation therapy. The methods used are appropriate to the aims of the study, they well described, and necessary controls are included. The conclusion that the lncRNA lncGRS-1 is a glioma-specific therapeutic target, is for the most part, adequately supported by the data presented. The authors provide sufficient details to allow replication and comparison with related analyses that have been performed. Overall, this study represents a significant advance over previously published studies and the paper is of broad interest to others in the field of cancer biology and long noncoding RNAs. The following concerns should be addressed before publication in Genome Biology.

1. On line 96, the authors start taking about lncGRS-1. Please provide more detail such as annotated name, chromosome location, etc for lncGRS-1 instead of providing these details later in the ms.

2. RNA-FISH in Figure 3 should also be done after knockdown of lncGRS-1 with CRISPRi or ASOs, to make sure that the nuclear foci correspond to this lncRNA.

3. What is the transfection efficiency of normal and GBM cells used for functional assays in this study? This can be determined by knocking down a protein coding gene such as GADPH or p53 and performing qRT-PCR. Do the primers for qRT-PCR for ASOs targeting lncGRS-1 span the binding site where the ASOs bind because it could be that that ASOs bind to the lncGRS-1 transcript post lysis and inhibit the qRT-PCR reaction.

4. Knockdown of lncGRS-1 did not alter proliferation in NHA (Figure 5). It would be good to show that knockdown of an essential gene alter viability in NHA as well as the GBM lines used in this study.

5. For Figure 5, the authors show volcano plots and correlative data for the genes that are differentially expressed upon knockdown of lncGRS-1. It is convincing because only 17 genes changed in NHA. However, the top 5 GO terms for the upregulated and downregulated genes is different in the U87 and SF8628 cells? It is suggested that the authors

6. It appears (in GTEX Portal), that lncGRS-1 is expressed in many normal human tissues. In RNA-seq in CCLE, is it expressed at higher levels in cell lines derived from some cancer types vs others? What happens to cell viability if lncGRS-1 is knocked down in a non-GBM cell line that expresses high levels of this lncRNA?

7. Given that the authors observed overrepresentation of the p53 pathway in the RNA-seq after knockdown of lncGRS-1, what is the effect of p53 and p21 mRNA and protein levels in this experiment? Does concurrent knockdown of p53 and lncGRS-1, rescue the proliferation to control cells? Along the same lines, what is the effect on p53BP1 foci and gamma H2AX when the lncGRS-1 knockdown cells are treated with radiation?

8. Transfection of A-MBOs with ASOs against lncGRS-1 showed effective knockdown but it appears from the smRNA-FISH, that lncGRS-1 is cytoplasmic in these cells? If this indeed is the case, it is a concern because it makes a mess of the potential mechanism proposed by the authors.

9. Line 562 and elsewhere, change107 to 107.

**Reviewer 2**

**Are you able to assess all statistics in the manuscript, including the appropriateness of statistical tests used?**

No, I do not feel adequately qualified to assess the statistics.

**Comments to author:**

Liu et al. identified potential lncRNA targets that sensitized cells to radiation utilizing a CRISPR interference (CRISPRi) screening in the U87 GBM cell line. From the screen, they chose lncGRS-1 as the top candidate. The authors show that knockdown of lncGRS-1 inhibited the growth and proliferation of primary adult and pediatric glioma cells, but not the viability of astrocytes. Additionally, the authors show that antisense oligonucleotides (ASOs) targeting lncGRS-1 decreased tumor growth and sensitized glioma cells to radiation. The major finding of this study is identification of lncGRS-1 as a potential radiation sensitizing target, which is potentially interesting and important to the field of radiation therapy of GBM. However, more evidence is needed to support the major finding that lncGRS-1 is a radiation sensitizer in vitro and in vivo. In addition, there is no mechanism described that would explain the lncGRS-1 phenotype. Therefore, this work needs further development and requires significant revision.

Specific concerns:

1. Fig 1b and 1c demonstrate a good example of a radiation sensitizer target (positive control); however, Fig3e,f,g and h do not show similar results to support that lncGRS-1 is a radiation sensitizer. These data suggest lncGRS-1 inhibition itself induced the effects no matter whether radiation is delivered or not. Combining lncGRS-1 inhibition with any other treatment might show similar results, suggesting this is not a radiation-specific phenomenon.

2. lncGRS-1 was prioritized based on the top "sensitizer score" (Fig 2d), which means a high ratio of the radiation modifier screen score in irradiated cells to the growth screen score in non-irradiated cells. However, Fig 4d demonstrates that lncGRS-1 KD cells were almost completely unable to proliferate over 15 days (especially for ASO #2), which indicates lncGRS-1 is more like an essential gene with high growth screen score rather than a "sensitizer".

3. The "competitive growth assay" is shown in Fig1 b-c and some other figures but no description is provided of how the experiment was performed.

4. In Fig 1b and Fig 3f and h, it's unclear when (i.e. what days) the fractioned radiation was delivered. This needs to be included (perhaps on the x-axis).

5. In Fig 5d, are there a common set of genes regulated by lncGRS-1 between U87 and SF8628? If so, are these genes related to DNA damage response?

6. To supporting the major finding, in vivo experiments should be performed to determine the radiation sensitizing effects of lncGRS-1 knockdown or ASOs targeting.

7. From the RNAseq shown in Fig S1, what's the dominant expression pattern of lncGRS-1?

8. Mechanistically, Fig S5 shows the downregulation of the G2 checkpoint pathway in U87 and cell cycle pathway in SF8628. Does lncGRS-1 inhibition affect the radiation-induced cell cycle arrest? How is the cell cycle pathway regulated by lncGRS-1?

9. In Fig S6d, is the sphere shown for different days the same sphere for each treatment condition? For example, Day 8 and Day18 of ASO control look very different.

10. Please define "Radiation Phenotype Score" in Fig2b and "Normalized Radiation Phenotype" in Fig S2c? The other "scores" used in the manuscript are also hard to understand, more information would be very helpful for understanding the analytic approach.

**Authors Response**

**Point-by-point responses to the reviewers’ comments:**

*RESPONSE TO REVIEWERS*

*Thank you for your helpful and insightful comments regarding our manuscript, “CRISPRi-based radiation modifier screen identifies long non-coding RNA therapeutic targets in glioma,” We have performed a number of new experiments and data analyses to support our findings and enhance our conclusions. With this new data and changes to the text, we believe that the paper has been greatly improved.*

*Below, we have responded to all comments, and each reviewer is addressed in full separately. We hope with the additional clarity that these experiments provide, you will find our manuscript suitable for publication in Genome Biology.*

*The new data can be found in the following figures:*

*Figure 5d*

*Figure 6b*

*Figure S1b*

*Figure S4a,b,c,h,i*

*Figure S5a,b,d,e,f,g,h*

*Figure S6a*

*Thank you again for your time and consideration.*

Reviewer #1:

Using Genome-wide CRISPRi screening, this elegant study identifies the lncRNA lncGRS-1 as a glioma-specific therapeutic target and establishes an approach to rapidly identify novel therapeutic targets in the vast non-coding genome to enhance the radiation therapy. The methods used are appropriate to the aims of the study, they well described, and necessary controls are included. The conclusion that the lncRNA lncGRS-1 is a glioma-specific therapeutic target, is for the most part, adequately supported by the data presented. The authors provide sufficient details to allow replication and comparison with related analyses that have been performed. Overall, this study represents a significant advance over previously published studies and the paper is of broad interest to others in the field of cancer biology and long noncoding RNAs. The following concerns should be addressed before publication in Genome Biology.

*We thank the reviewer for their positive evaluation of the manuscript.*

1. On line 96, the authors start taking about lncGRS-1. Please provide more detail such as annotated name, chromosome location, etc for lncGRS-1 instead of providing these details later in the ms.

*We have added genome annotation information in the first mention of lncGRS-1 (CTC-338M12.4 located on chromosome 5 q35.3).*

2. RNA-FISH in Figure 3 should also be done after knockdown of lncGRS-1 with CRISPRi or ASOs, to make sure that the nuclear foci correspond to this lncRNA.

*We performed RNA-FISH in SF8628 cells for lncGRS-1 following knockdown and observed depletion of nuclei foci corresponding to lncGRS-1. These data are included as Figure S4a.*

3. What is the transfection efficiency of normal and GBM cells used for functional assays in this study? This can be determined by knocking down a protein coding gene such as GADPH or p53 and performing qRT-PCR. Do the primers for qRT-PCR for ASOs targeting lncGRS-1 span the binding site where the ASOs bind because it could be that that ASOs bind to the lncGRS-1 transcript post lysis and inhibit the qRT-PCR reaction.

*Knockdown of p53 demonstrated 99% knockdown efficiency using ASOs, suggesting a high degree of transfection efficiency (ASO concentration 25 nM) (included now in Figure S4b).*

*The qPCR primers targeting lncGRS-1 span the exon 2-3 junction of lncGRS-1 transcript, which we now depict in the schematic in Figure S4c. These primers do span the targeting site of lncGRS-1 ASO #2 but NOT #1. Since both ASO’s result in robust knockdown of lncGRS-1 (Figure 4c, 5a, S4d-g), this does not support the possibility of post-lysis knockdown of lncGRS-1 affecting results as suggested by the reviewer.*

4. Knockdown of lncGRS-1 did not alter proliferation in NHA (Figure 5). It would be good to show that knockdown of an essential gene alter viability in NHA as well as the GBM lines used in this study.

*Knockdown of essential gene POLA1 (DNA polymerase Alpha 1) resulted in decreased proliferation of both NHA cells and U87 GBM cells. These data have been added in Figure S4h,i.*

5. For Figure 5, the authors show volcano plots and correlative data for the genes that are differentially expressed upon knockdown of lncGRS-1. It is convincing because only 17 genes changed in NHA. However, the top 5 GO terms for the upregulated and downregulated genes is different in the U87 and SF8628 cells? It is suggested that the authors [sic]

*We acknowledge that the top 5 GO terms for differentially expressed genes are different in U87 and SF8628 cells. However, genome-wide quantitative comparison of genes that are differentially expressed in either U87 or DIPG SF8628 cells demonstrate a positive correlation in gene expression changes between the disparate cell lines (Pearson R = 0.667). This result has been added to Figure S5d.*

6. It appears (in GTEX Portal), that lncGRS-1 is expressed in many normal human tissues. In RNA-seq in CCLE, is it expressed at higher levels in cell lines derived from some cancer types vs others? What happens to cell viability if lncGRS-1 is knocked down in a non-GBM cell line that expresses high levels of this lncRNA?

*Bioinformatics analysis of lncGRS-1 expression in the CCLE database (Ghandi et al. 2019, Nature) reveals robust expression across numerous cell types. We knocked down lncGRS-1 in the HeLa cervical cancer cell line that expresses high levels of lncGRS-1 (TPM = 4.38) and observed no appreciable proliferation defect in these cells using the same sgRNAs that were used in GBM cells. These data are included in Figure S5a,b.*

7. Given that the authors observed overrepresentation of the p53 pathway in the RNA-seq after knockdown of lncGRS-1, what is the effect of p53 and p21 mRNA and protein levels in this experiment? Does concurrent knockdown of p53 and lncGRS-1, rescue the proliferation to control cells? Along the same lines, what is the effect on p53BP1 foci and gamma H2AX when the lncGRS-1 knockdown cells are treated with radiation?

*We observed decreased levels of p53 transcripts (TP53) and increased p21 transcripts (CDKN1A) after knockdown of lncGRS-1, which are corroborated with protein level western blots. The increase in p21 levels may in part underlie the decrease in cell cycle progression, which was a result obtained from an experimental suggestion of Reviewer 2 (Figure 5d). Although seemingly paradoxical that p53 levels decrease while p53 effectors and p21 are upregulated, we note that this could be explained by activation of the p53 signaling pathway that is independent of absolute p53 levels, such as through nuclear localization and phosphorylation. We also consider the possibility of p53-independent activation of p21 which has been described previously (Huo et. al 2014).*

*Interestingly, concurrent knockdown of p53 and lncGRS-1 does not rescue the proliferation caused by lncGRS-1 knockdown. Instead, double knockdown led to further decrease in proliferation in U87 cells. We postulate that elucidating the mechanistic relationship between lncGRS-1 and p53 will require significantly more experiments and is beyond the scope of this study, and therefore we have focused on the p21 results for this manuscript. These data are now included as Figure S5e,f.*

*Regarding p53BP1 and gamma H2AX foci, lncGRS-1 knockdown resulted in increased p53BP1 foci in the absence of radiation. Interestingly, gamma H2AX foci induced by radiation were potentiated by knockdown of lncGRS-1, but lncGRS-1 knockdown in the absence of radiation did not generate additional gamma H2AX foci. These data are included in Figure S5g,h.*

*Taken together, these results indicate that lncGRS-1 plays roles in the regulation of cell cycle and DNA damage response. We thank both reviewers for their experimental suggestions, as these data provide early insights into the mechanism(s) of lncGRS-1 function in glioma.*

8. Transfection of A-MBOs with ASOs against lncGRS-1 showed effective knockdown but it appears from the smRNA-FISH, that lncGRS-1 is cytoplasmic in these cells? If this indeed is the case, it is a concern because it makes a mess of the potential mechanism proposed by the authors.

*We have performed additional single molecule FISH demonstrating the nuclear localization of lncGRS-1 in A-MBOs with and without ASO knockdown (Figure 6b). We then quantified the number of nuclear foci of lncGRS-1 using RNA-FISH following knockdown in A-MBOs and demonstrate depletion of lncGRS-1 foci corresponding to knockdown. These data are included as new Figure S6a.*

9. Line 562 and elsewhere, change107 to 107.

*We have clarified the notations here and changed 10^7 to “10 million” for clarity.*

Reviewer #2:

Liu et al. identified potential lncRNA targets that sensitized cells to radiation utilizing a CRISPR interference (CRISPRi) screening in the U87 GBM cell line. From the screen, they chose lncGRS-1 as the top candidate. The authors show that knockdown of lncGRS-1 inhibited the growth and proliferation of primary adult and pediatric glioma cells, but not the viability of astrocytes. Additionally, the authors show that antisense oligonucleotides (ASOs) targeting lncGRS-1 decreased tumor growth and sensitized glioma cells to radiation. The major finding of this study is identification of lncGRS-1 as a potential radiation sensitizing target, which is potentially interesting and important to the field of radiation therapy of GBM. However, more evidence is needed to support the major finding that lncGRS-1 is a radiation sensitizer in vitro and in vivo. In addition, there is no mechanism described that would explain the lncGRS-1 phenotype. Therefore, this work needs further development and requires significant revision.

For the readership of Genome Biology, we believe that the focus and strengths of this manuscript to be: 1) the successful genome-scale exploration of lncRNAs for potential targets in a clinically-oriented context, 2) an example of how CRISPRi can be used to search for modifiers of standard-of-care therapy (fractionated radiation, in this case), and 3) how “mature” brain organoids (a novel organoid-based model of glioma) can be used to further study a screen-identified target. We believe that this workflow will be broadly useful to the study of many different types of cancer. Our follow-up studies of lncGRS-1 were presented as an example of the utility of this workflow and is not intended to be a full characterization of this target. We put forth that detailed mechanism of lncGRS-1 is beyond the scope of this manuscript, however, in response to specific experimental requests of both reviewers, we have done some work in this area and now include these data in the revision.

Specific concerns:

1. Fig 1b and 1c demonstrate a good example of a radiation sensitizer target (positive control); however, Fig3e,f,g and h do not show similar results to support that lncGRS-1 is a radiation sensitizer. These data suggest lncGRS-1 inhibition itself induced the effects no matter whether radiation is delivered or not. Combining lncGRS-1 inhibition with any other treatment might show similar results, suggesting this is not a radiation-specific phenomenon.

*We agree that lncGRS-1 does not solely function as a radiation-specific modifier. lncGRS-1 falls into a class of clinical radiation sensitizer targets that are also cytotoxic on their own. For instance, the chemotherapeutic agent cisplatin is considered to be a radiation sensitizer and is also cytotoxic on its own. Of note, in our radiation-modifier screen, we find buffering hits (right side of the plot in Figure 2b), in addition to hits that are radiation sensitizer hits.*

2. lncGRS-1 was prioritized based on the top "sensitizer score" (Fig 2d), which means a high ratio of the radiation modifier screen score in irradiated cells to the growth screen score in non-irradiated cells. However, Fig 4d demonstrates that lncGRS-1 KD cells were almost completely unable to proliferate over 15 days (especially for ASO #2), which indicates lncGRS-1 is more like an essential gene with high growth screen score rather than a "sensitizer".

*We acknowledge the dual nature of the function of lncGRS-1 as having properties of both an essential gene and also as a radiation sensitizer and have amended the Discussion section to reflect this.*

3. The "competitive growth assay" is shown in Fig1 b-c and some other figures but no description is provided of how the expriment was performed.

*We have clarified the “competitive growth assay” by making the terminology consistent, that is, “internally controlled growth assay,” which is now described in the methods section and also cited for additional reference as follows: “Internally controlled CRISPRi growth assays were performed as previously described, briefly, by partially infecting a population of cells stably expressing dCas9-KRAB with a fluorescently labeled sgRNA expression vector and tracking the sgRNA-infected population over time using flow cytometry relative to the non-infected population (Liu et al. 2017, Science).”*

4. In Fig 1b and Fig 3f and h, it's unclear when (i.e. what days) the fractioned radiation was delivered. This needs to be included (perhaps on the x-axis).

*We have amended the figures 1b and 3f and h to indicate clearly on the x-axis the days on which fractionated radiation were delivered. The difference in radiation delivery schedule between 3f and 3h was a consequence of the additional 4 days of puromycin cell selection and recovery that occurred in the experiment in purified cell population indicated in 3h that was not required in the internally controlled growth assay performed in 3f. Therefore, radiation was initiated at identical time intervals following the transduction of sgRNA expressing lentivirus.*

5. In Fig 5d, are there a common set of genes regulated by lncGRS-1 between U87 and SF8628? If so, are these genes related to DNA damage response?

*We performed genome-wide quantitative comparison of genes that are differentially expressed in either U87 or DIPG SF8628 cells, and we demonstrate a positive correlation in gene expression changes between the two cell lines (Pearson R = 0.667; New Figure S5d). Among the common upregulated genes are the canonical cellular stress response activators ATF3 and XBP1. We postulate that DNA damage response does occur at the post-transcriptional level, and we demonstrate that lncGRS-1 knockdown potentiates the accumulation of gamma H2AX foci when cells are exposed to radiation (New Figure S5h).*

6. To supporting the major finding, in vivo experiments should be performed to determine the radiation sensitizing effects of lncGRS-1 knockdown or ASOs targeting.

*We fully appreciate this reviewer’s sentiment and agree that in vivo validation data would strengthen our studies of lncGRS-1. We discussed with the Editor the amount of time required for the completion of such studies. Briefly, we would need to first determine the appropriate xenograft cell transplant “dose” as well as the dose of radiation (total dose and fractionation plan) that models the “partial” treatment effect observed in human patients. This would need to be determined empirically. If the radiation dose is too high, tumor growth will be completely abolished, and if the radiation dose is too low, there will be little radiation effect to modify. Such experimental parameters would need to be established before trying this in combination with in vivo lncGRS-1 knockdown methods, which would also need to be developed empirically.*

*Given the broad, general interest in exploring lncRNAs as therapeutic targets, the use of CRISPR-based technologies for functional screens of the noncoding genome, and new organoid-based models of tumor growth, the Editor suggested that we instead focus on the comments that relate to experimental controls. Without in vivo studies, we believe this manuscript will be of broad interest to the readership of Genome Biology. To help address this reviewer’s comment, in the Discussion section, we now mention that future in vivo validation studies of lncGRS-1 should be pursued to further develop this particular lncRNA for potential clinical development.*

7. From the RNAseq shown in Fig S1, what's the dominant expression pattern of lncGRS-1?

*lncGRS-1 is downregulated following radiation treatment in a dose dependent fashion. We have included this result in Figure S1b.*

8. Mechanistically, Fig S5 shows the downregulation of the G2 checkpoint pathway in U87 and cell cycle pathway in SF8628. Does lncGRS-1 inhibition affect the radiation-induced cell cycle arrest? How is the cell cycle pathway regulated by lncGRS-1?

*Knockdown of lncGRS-1 results in decreased G2 and M phase representation in populations of U87 cells by flow cytometry analysis, supporting the hypothesis that cells without lncGRS-1 are diminished in progression through the cell cycle (new Figure 5d), as would be expected following radiation. While it is still unclear how lncGRS-1 regulates cell cycle progression, we found upregulation of CDKN1A following lncGRS-1 knockdown, which may partially explain the phenotype observed (new Figure S5e).*

9. In Fig S6d, is the sphere shown for different days the same sphere for each treatment condition? For example, Day 8 and Day18 of ASO control look very different.

*The organoid experiment performed in Fig S6d (new Figure S6e) involves serial microscopic imaging of individual organoids isolated within individual wells of a 96 well dish. Therefore, each image represents the same organoid over time. We acknowledge that the morphology of these organoids may change slightly over time when invaded by tumor cells. We have amended the manuscript to clarify this in both results and method sections as follows: “RFP labeled U87 cells were seeded to the surface of individual AN-MBOs isolated across multiple wells of a 96 well dish. Tumors grew invasively (Fig. S6c) and progressively larger when treated with negative control ASOs and no radiation when tracked using serial microscopic imaging (Fig. S6e).”*

10. Please define "Radiation Phenotype Score" in Fig2b and "Normalized Radiation Phenotype" in Fig S2c? The other "scores" used in the manuscript are also hard to understand, more information would be very helpful for understanding the analytic approach.

*We apologize for the confusion. We have clarified the phenotypes and scores listed in the manuscript as follows. Phenotype refers to “the relative log2 enrichment of barcodes in the final timepoint divided by the enrichment of barcodes at the initial timepoint,” which we make explicit in the results, methods, and figure legend sections. Where phenotypes are listed as “z-standardized,” as in Fig S2c, standardization was performed by normalizing by the standard deviation of negative control genes’ phenotypes, as is now listed in the methods and figure legends section.*

*Whereas “screen score,” of which there are 2 indicated in the manuscript: growth screen score and radiation modifier screen score, are defined as “the average phenotype of the top three sgRNAs against a given gene multiplied by the negative log10(Mann-Whitney-U p value) for that gene,” which is now described clearly in the main text, methods, and figure legends section. The difference between growth screen score and radiation screen score is based on whether the results were obtained from the growth CRISPRi screen (without radiation) or from the radiation modifier CRISPRi screen (with radiation). The final remaining “score,” the “sensitizer score,” is defined as “the ratio of the radiation modifier screen score in irradiated cells to the growth screen score in non-irradiated cells,” as indicated in the main text, is also now also included in the methods and figure legends for clarity.*

*In addition, we have clarified all instances of scores and phenotypes such that “phenotype” and “score” are not used in the same phrase and potentially confused.*

**Second round of review**

**Reviewer 1**

The authors have addressed my concerns
